# Supplementary material for: Hiss and tell: What influences venom yields of India’s big four snakes?
Source: PLoS Negl Trop Dis. 2025 Nov 3;19(11):e0013676. doi: 10.1371/journal.pntd.0013676 (PMC12591399; doi:10.1371/journal.pntd.0013676)
Supplement: S1 File — This file provides the complete R code used for analysing venom yield data, including the Shapiro-Wilk normality test, effect-size calculations, median absolute deviation (MAD), and coefficient of variation (CoV). (DOCX) [file pntd.0013676.s001.docx]

**S1 File. R script and normality-test results.** This file provides the complete R code used for analysing venom yield data, including the Shapiro-Wilk normality test, effect-size calculations, median absolute deviation (MAD), and coefficient of variation (CoV).

library(ggplot2)
library(dplyr)

library(stringr)
library(FSA)

## ===== Load data and normalize names =====
 data_all <- read.csv("Big_4.csv", stringsAsFactors = FALSE)
 head(data_all)

if (!"Scientific_name" %in% names(data_all) && "Scientific.name" %in% names(data_all)) {
 data_all <- dplyr::rename(data_all, Scientific_name = Scientific.name)
 }
 if (!"Dry_wt_mg" %in% names(data_all) && "Dry.wt..mg." %in% names(data_all)) {
 data_all <- dplyr::rename(data_all, Dry_wt_mg = `Dry.wt..mg.`)
 }

 data_all <- data_all %>%
 mutate(
 Scientific_name = str_squish(Scientific_name),
 Dry_wt_mg = as.numeric(Dry_wt_mg)
 )

***## ===== Normality (Shapiro–Wilk per species) =====***

shapiro_results <- data_all **%>% group_by**(Scientific_name) **%>%**

**summarise**(

n = **sum**(**!is.na**(Dry_wt_mg)),

W = **if** (n **>=** 3 **&&** n **<=** 5000) **shapiro.test**(Dry_wt_mg)**$**statistic

**else** NA_real_,

shapiro_p = **if** (n **>=** 3 **&&** n **<=** 5000) **shapiro.test**(Dry_wt_mg)**$**p.value

**else** NA_real_,

.groups = "drop" ) **%>%**

**mutate**(normality = **ifelse**(**!is.na**(shapiro_p) **&** shapiro_p **>** 0.05, "Normal", "Not normal"))

**print**(shapiro_results)

## # A tibble: 5 × 5

## Scientific_name n W shapiro_p normality

## <chr> <int> <dbl> <dbl> <chr>

## 1 Bungarus caeruleus 41 0.869 2.31e- 4 Not normal

## 2 Daboia russelii 115 0.747 8.38e-13 Not normal

## 3 Echis carinatus 30 0.888 4.39e- 3 Not normal

## 4 Echis sochureki 8 0.880 1.90e- 1 Normal

## 5 Naja naja 144 0.823 6.97e-12 Not normal

##Visual Result########################################################

ggplot(data_all, aes(sample = Dry_wt_mg, color = normality)) +

stat_qq() +

stat_qq_line() +

facet_wrap(~ Scientific_name, scales = "free") +

theme_minimal() +

labs(title = "QQ Plot by Species with Normality Status")


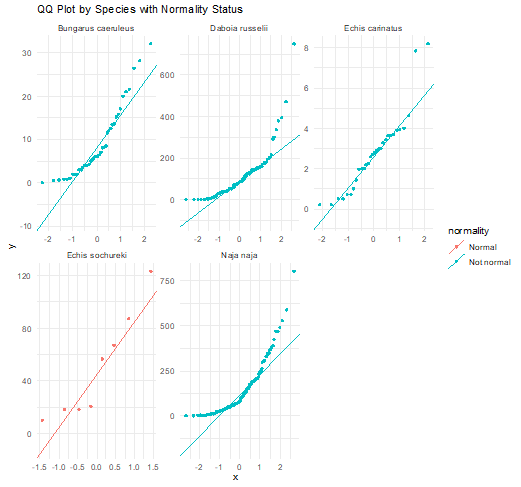

 ## ===== Quick medians =====
 meds <- data_all %>%
 group_by(Scientific_name) %>%
 summarise(median = median(Dry_wt_mg, na.rm = TRUE), .groups = "drop")

 ## ===== Normality (Shapiro–Wilk per species) =====
 shapiro_results <- data_all %>%
 group_by(Scientific_name) %>%
 summarise(
 n = sum(!is.na(Dry_wt_mg)),
 W = if (n >= 3 && n <= 5000) shapiro.test(Dry_wt_mg)$statistic else NA_real_,
 shapiro_p = if (n >= 3 && n <= 5000) shapiro.test(Dry_wt_mg)$p.value else NA_real_,
 .groups = "drop"
 ) %>%
 mutate(normality = ifelse(!is.na(shapiro_p) & shapiro_p > 0.05, "Normal", "Not normal"))

 print(shapiro_results)

## ===== Kruskal–Wallis + Dunn (BH) =====
 kw_result <- kruskal.test(Dry_wt_mg ~ Scientific_name, data = data_all)
 dunn_result <- dunnTest(Dry_wt_mg ~ Scientific_name, data = data_all, method = "bh")

dunn_res <- dunn_result$res
 ###=====Summary_stats============================
 summary_stats <- data_all %>%
 group_by(Scientific_name) %>%
 summarise(
 count = n(),
 mean = mean(Dry_wt_mg, na.rm = TRUE),
 median = median(Dry_wt_mg, na.rm = TRUE),
 sd = sd(Dry_wt_mg, na.rm = TRUE),
 min = min(Dry_wt_mg, na.rm = TRUE),
 q1 = quantile(Dry_wt_mg, 0.25, na.rm = TRUE),
 q3 = quantile(Dry_wt_mg, 0.75, na.rm = TRUE),
 max = max(Dry_wt_mg, na.rm = TRUE)
 )

 # Nonparametric effect size (epsilon-squared)
 k <- nlevels(data_all$Scientific_name)
 n <- nrow(data_all)
 H <- as.numeric(kw_result$statistic)
 epsilon2 <- (H - k + 1) / (n - k)
 epsilon2 <- max(0, epsilon2) # guard against tiny negative due to rounding
 subtitle_txt <- sprintf("Kruskal–Wallis: \u03C7\u00B2 = %.3f (df=%d), p = %.4f \u2022 \u03B5\u00B2 = %.3f",
 H, as.integer(kw$parameter), kw$p.value, epsilon2)

#######################################Naja_Naja#############
 dat_Naja <- read.csv("Naja_Biogeography.csv", stringsAsFactors = FALSE)

# --- Kruskal–Wallis across Biogeography ---
 kw <- kruskal.test(Dry_wt ~ Biogeography, data = dat_Naja)
 print(kw)

# Nonparametric effect size (epsilon-squared)
 k <- nlevels(dat_Naja$Biogeography)
 n <- nrow(dat_Naja)
 H <- as.numeric(kw$statistic)
 epsilon2 <- (H - k + 1) / (n - k)
 epsilon2 <- max(0, epsilon2) # guard against tiny negative due to rounding
 subtitle_txt <- sprintf("Kruskal–Wallis: \u03C7\u00B2 = %.3f (df=%d), p = %.4f \u2022 \u03B5\u00B2 = %.3f",
 H, as.integer(kw$parameter), kw$p.value, epsilon2)

#######################################Daboia_russelii####

dat_Dab <- read.csv("Daboia_Biogeography.csv", stringsAsFactors = FALSE)

# --- Kruskal–Wallis across Biogeography ---
 kw <- kruskal.test(Dry_wt ~ Biogeography, data = dat_Dab)
 print(kw)

##
 ## Kruskal-Wallis rank sum test
 ##
 ## data: Dry_wt by Biogeography
 ## Kruskal-Wallis chi-squared = 13.05, df = 4, p-value = 0.01103

# Nonparametric effect size (epsilon-squared) for KW
 k <- nlevels(dat_Dab$Biogeography)
 n <- nrow(dat_Dab)
 H <- as.numeric(kw$statistic)
 epsilon2 <- (H - k + 1) / (n - k)
 epsilon2 <- max(0, epsilon2) # guard against tiny negative due to rounding
 subtitle_txt <- sprintf("Kruskal–Wallis: \u03C7\u00B2 = %.3f (df=%d), p = %.4f \u2022 \u03B5\u00B2 = %.3f",
 H, as.integer(kw$parameter), kw$p.value, epsilon2)

#######################################Bungarus_caeruleus######

dat_Bun <- read.csv("Bungarus_caeruleus_Biogeography.csv", stringsAsFactors = FALSE)

# --- Kruskal–Wallis across Biogeography ---
 kw <- kruskal.test(Dry_wt ~ Biogeography, data = dat_Bun)
 print(kw)

# Nonparametric effect size (epsilon-squared) for KW
 k <- nlevels(dat_Bun$Biogeography)
 n <- nrow(dat_Bun)
 H <- as.numeric(kw$statistic)
 epsilon2 <- (H - k + 1) / (n - k)
 epsilon2 <- max(0, epsilon2) # guard against tiny negative due to rounding
 subtitle_txt <- sprintf("Kruskal–Wallis: \u03C7\u00B2 = %.3f (df=%d), p = %.4f \u2022 \u03B5\u00B2 = %.3f",
 H, as.integer(kw$parameter), kw$p.value, epsilon2)

#######################################Echis_carinatus######

dat_Echis <- read.csv("Echis_Biogeography.csv", stringsAsFactors = FALSE)

# --- Kruskal–Wallis across Biogeography ---
 kw <- kruskal.test(Dry_wt ~ Biogeography, data = dat_Echis)

# Nonparametric effect size (epsilon-squared) for KW
 k <- nlevels(dat_Echis$Biogeography)
 n <- nrow(dat_Echis)
 H <- as.numeric(kw$statistic)
 epsilon2 <- (H - k + 1) / (n - k)
 epsilon2 <- max(0, epsilon2) # guard against tiny negative due to rounding
 subtitle_txt <- sprintf("Kruskal–Wallis: \u03C7\u00B2 = %.3f (df=%d), p = %.4f \u2022 \u03B5\u00B2 = %.3f",
 H, as.integer(kw$parameter), kw$p.value, epsilon2)

####CV and MAD #####################################################

data_all <- read.csv("Big_4.csv", stringsAsFactors = FALSE)

head(data_all)

if (!"Scientific_name" %in% names(data_all) && "Scientific.name" %in% names(data_all)) {
 data_all <- dplyr::rename(data_all, Scientific_name = Scientific.name)
 }
 if (!"Dry_wt_mg" %in% names(data_all) && "Dry.wt..mg." %in% names(data_all)) {
 data_all <- dplyr::rename(data_all, Dry_wt_mg = `Dry.wt..mg.`)
 }

 data_all <- data_all %>%
 mutate(
 Scientific_name = str_squish(Scientific_name),
 Dry_wt_mg = as.numeric(Dry_wt_mg)
 )
 # ===== Per-species summary stats =====
 summary_by_species <- data_all %>%
 group_by(Scientific_name) %>%
 summarise(
 n = sum(!is.na(Dry_wt_mg)),
 median_dry_mg = median(Dry_wt_mg, na.rm = TRUE),
 mean_dry_mg = mean(Dry_wt_mg, na.rm = TRUE),
 sd_dry_mg = sd(Dry_wt_mg, na.rm = TRUE),
 # raw MAD around the median (no scaling)
 mad_raw_dry_mg = mad(Dry_wt_mg, center = median(Dry_wt_mg, na.rm = TRUE),
 constant = 1, na.rm = TRUE),
 cv_percent = ifelse(mean_dry_mg == 0 | is.na(mean_dry_mg),
 NA_real_,
 100 * sd_dry_mg / mean_dry_mg),
 .groups = "drop"
 )

## CV% barplot
 ggplot(summary_by_species, aes(x = Scientific_name, y= cv_percent)) + geom_col (fill = "lightblue") +
 geom_text(aes(label = round(cv_percent, 1)), vjust = -0.5) +
 labs (
 title = "Intraspecific variability in venom yield",
 x = "Species",
 y = "Coefficient of Variation (%)"
 )+
 theme_minimal(base_size = 15)

### MAD
 ggplot(summary_by_species, aes(x = Scientific_name, y= mad_raw_dry_mg)) + geom_col (fill = "lightblue") +
 geom_text(aes(label = round(mad_raw_dry_mg, 1)), vjust = -0.5) +
 labs (
 title = "Intraspecific variability in venom yield",
 x = "Species",
 y = "MAD"
 )+
 theme_minimal(base_size = 15)
