# Supplementary material for: Hiss and tell: What influences venom yields of India’s big four snakes?
Source: PLoS Negl Trop Dis. 2025 Nov 3;19(11):e0013676. doi: 10.1371/journal.pntd.0013676 (PMC12591399; doi:10.1371/journal.pntd.0013676)
Supplement: S1 Fig — The image depicts the Naja naja venom extraction procedure, in which the snake is gently coaxed to bite a membrane stretched over a sterile beaker, allowing for the collection of venom without the need for external pressure. Photo credit: Ajinkya Unawane. (DOCX) [file pntd.0013676.s002.docx]

**S1 Fig.** Snake venom extraction


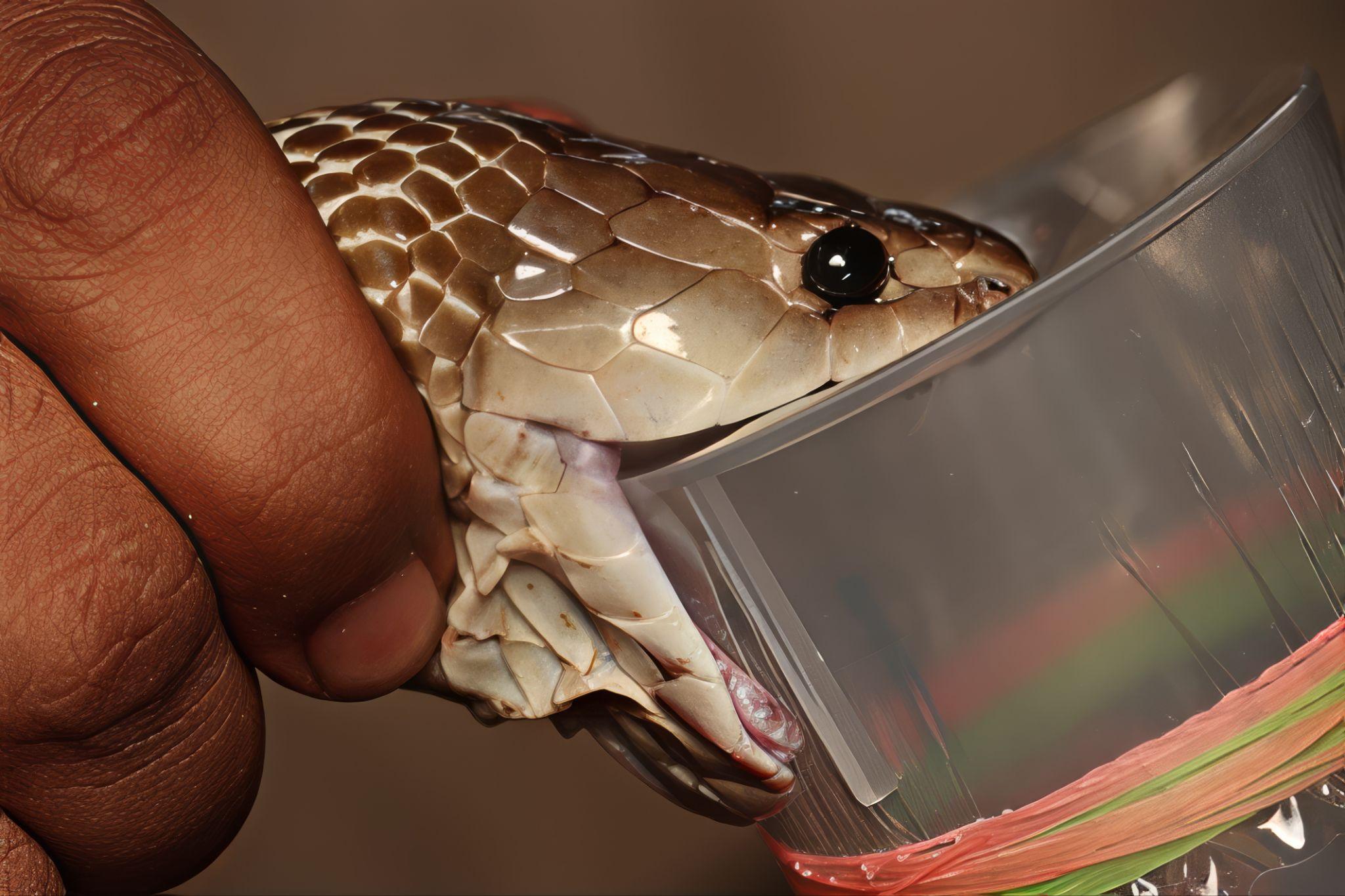


The image depicts the *Naja naja* venom extraction procedure, in which the snake is gently coaxed to bite a membrane stretched over a sterile beaker, allowing for the collection of venom without the need for external pressure. Photo credit: Ajinkya Unawane.
