# Supplementary material for: Hiss and tell: What influences venom yields of India’s big four snakes?
Source: PLoS Negl Trop Dis. 2025 Nov 3;19(11):e0013676. doi: 10.1371/journal.pntd.0013676 (PMC12591399; doi:10.1371/journal.pntd.0013676)

S2 Fig. Comparison of venom yields between captive and wild snakes.

In the figure, venom yield (measured in mg, dry weight) was compared between previously published data and the findings from the current study. For N. naja, D. russelii, and B. caeruleus (panel A), as well as E. c. carinatus and E. c. sochureki (panel B), venom yields from captive snakes, as reported by Whitaker and Whitaker (2012) [21] and Tumbare and Khadilkar (2004) [9], were assessed alongside the venom yields collected from wild snakes in this study. The data are expressed as mean ± SEM (represented by error bars), with non-significant comparisons indicated by "ns".
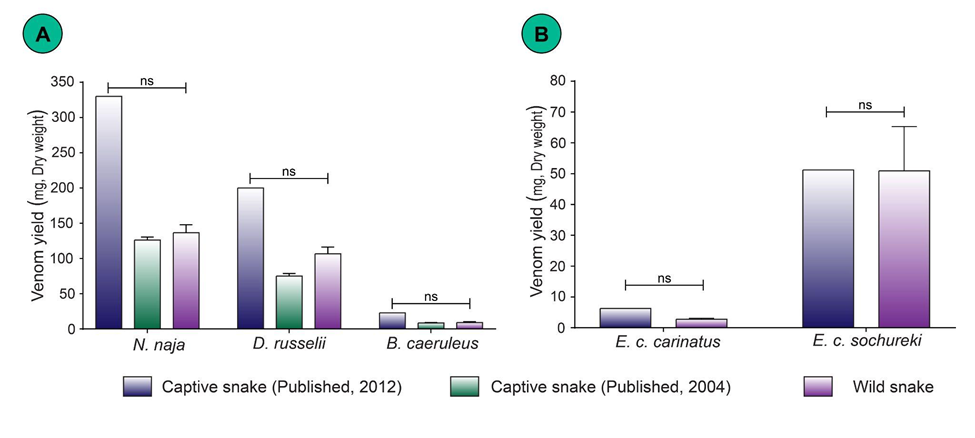

Supplement: S2 Fig — In the figure, venom yield (measured in mg, dry weight) was compared between previously published data and the findings from the current study. For N. naja, D. russelii, and B. caeruleus (panel A), as well as E. c. carinatus and E. c. sochureki (panel B), venom yields from captive snakes, as reported by Whitaker and Whitaker (2012) and Tumbare and Khadilkar (2004), were assessed alongside the venom yields collected from wild snakes in this study. The data are expressed as mean ± SEM (represented by error bars), with non-significant comparisons indicated by “ns”. (DOCX) [file pntd.0013676.s003.docx]
