# Supplementary material for: Hiss and tell: What influences venom yields of India’s big four snakes?
Source: PLoS Negl Trop Dis. 2025 Nov 3;19(11):e0013676. doi: 10.1371/journal.pntd.0013676 (PMC12591399; doi:10.1371/journal.pntd.0013676)
Supplement: S1 Table — This table summarises venom yield statistics for the ‘big four’ Indian snakes: N. naja, D. russelii, B. caeruleus, and E. c. carinatus (including its subspecies E. c. sochureki). The data are presented as sample size (N), mean ± SEM (standard error of the mean), median, standard deviation (SD), minimum venom yield (min), first quartile (Q1), third quartile (Q3), and maximum venom yield (max). For some samples, locality, gender, and/or age were not recorded. (DOCX) [file pntd.0013676.s004.docx]

**S1 Table**. Venom yield statistics for the ‘big four’ Indian snakes.

| **Species** | **Category** | **Subcategory** | **N** | **Mean ± SEM** | **Median** | **SD** | **Min** | **Q1** | **Q3** | **Max** |
| --- | --- | --- | --- | --- | --- | --- | --- | --- | --- | --- |
| ***N. naja*** | **Venom yield** | - | 144 | 136.10 ± 11.16 | 83.64 | 133.90 | 0.50 | 41.49 | 194.00 | 800.40 |
|  | **Biogeography** | Semi-arid | 3 | 188.50 ± 94.90 | 194.00 | 164.40 | 21.40 | 108.00 | 272.00 | 350.00 |
|  |  | Deserts | 3 | 116.60 ± 58.15 | 151.80 | 100.70 | 3.00 | 77.40 | 173.00 | 195.00 |
|  |  | Coast | 20 | 151.50 ± 28.39 | 130.80 | 127.00 | 10.00 | 57.00 | 204.00 | 468.70 |
|  |  | Gangatic Plains | 11 | 111.10 ± 18.89 | 111.60 | 62.64 | 33.00 | 58.50 | 156.00 | 214.00 |
|  |  | Deccan Peninsula | 55 | 145.30 ± 18.30 | 79.30 | 135.70 | 9.13 | 45.70 | 205.00 | 528.30 |
|  |  | Western Ghats | 51 | 123.00 ± 20.92 | 73.00 | 149.40 | 0.50 | 31.20 | 181.00 | 800.40 |
|  | **Gender** | Male | 81 | 141.00 ± 15.00 | 103.00 | 139.00 | 0.50 | 54.00 | 201.0 | 800.40 |
|  |  | Female | 25 | 114.00 ± 23.00 | 63.00 | 117.00 | 3.20 | 29.80 | 150.00 | 378.00 |
|  | **Ontogeny** | Adult | 111 | 159.00 ± 13.00 | 125.00 | 133.00 | 9.10 | 60.90 | 208.00 | 800.40 |
|  |  | Subadult | 16 | 45.90 ± 8.20 | 47.60 | 32.80 | 3.00 | 25.30 | 59.90 | 131.00 |
|  |  | Juvenile | 10 | 10.00 ± 3.33 | 6.50 | 10.50 | 0.50 | 3.50 | 11.50 | 34.40 |
| ***D. russelii*** | **Venom yield** | - | 115 | 106.60 ± 9.61 | 86.14 | 103.10 | 0.50 | 40.54 | 144.20 | 749.00 |
|  | **Biogeography** | Semi-arid | 2 | 83.67 ± 31.15 | 83.67 | 44.05 | 52.52 | 68.10 | 99.20 | 114.80 |
|  |  | Coast | 3 | 47.27 ± 5.23 | 50.70 | 9.05 | 37.00 | 43.80 | 52.40 | 54.10 |
|  |  | Gangatic Plains | 14 | 103.90 ± 41.97 | 28.00 | 157.00 | 1.00 | 3.20 | 92.00 | 470.00 |
|  |  | Deccan Peninsula | 54 | 131.80 ± 15.78 | 125.50 | 116.00 | 1.00 | 60.90 | 155.00 | 749.00 |
|  |  | Western Ghats | 42 | 80.38 ± 7.65 | 73.78 | 49.58 | 0.50 | 41.90 | 122.00 | 203.00 |
|  | **Gender** | Male | 27 | 85.32 ± 11.75 | 70.00 | 61.08 | 7.00 | 39.80 | 133.00 | 216.00 |
|  |  | Female | 48 | 107.90 ± 13.23 | 102.00 | 91.64 | 0.50 | 52.00 | 144.00 | 470.00 |
|  | **Ontogeny** | Adult | 92 | 118.10 ± 11.02 | 95.69 | 105.70 | 1.00 | 53.60 | 144.00 | 749.00 |
|  |  | Subadult | 7 | 39.97 ± 11.75 | 33.22 | 31.09 | 1.00 | 21.50 | 56.80 | 89.00 |
|  |  | Juvenile | 9 | 8.99 ± 4.93 | 3.00 | 14.81 | 0.50 | 2.00 | 7.50 | 47.40 |
| ***B. caeruleus*** | **Venom yield** | - | 41 | 8.95 ± 1.28 | 6.00 | 8.18 | 0.10 | 3.00 | 13.43 | 32.00 |
|  | **Biogeography** | Coast | 3 | 9.37 ± 3.41 | 8.40 | 5.91 | 4.00 | 6.20 | 12.00 | 15.70 |
|  |  | Gangatic Plains | 8 | 14.63 ± 2.55 | 12.94 | 7.22 | 5.99 | 10.20 | 20.20 | 26.30 |
|  |  | Deccan Peninsula | 14 | 9.59 ± 2.64 | 6.26 | 9.87 | 0.10 | 3.60 | 13.10 | 32.00 |
|  |  | Western Ghats | 16 | 5.79 ± 1.53 | 4.00 | 5.94 | 0.50 | 2.00 | 6.30 | 21.60 |
|  | **Gender** | Male | 15 | 9.62 ± 1.92 | 6.50 | 7.45 | 2.00 | 4.00 | 13.50 | 28.00 |
|  |  | Female | 12 | 8.63 ± 2.44 | 5.13 | 8.46 | 0.50 | 2.00 | 13.80 | 26.30 |
|  | **Ontogeny** | Adult | 40 | Not available | | | | | | |
|  |  | Subadult | 0 |  |  |  |  |  |  |  |
|  |  | Juvenile | 1 |  |  |  |  |  |  |  |
| ***E. c. carinatus*** | **Venom yield** | - | 30 | 2.76 ± 0.35 | 2.75 | 1.91 | 0.20 | 1.33 | 3.67 | 8.20 |
|  | **Biogeography** | Coast | 12 | 3.02 ± 0.61 | 3.46 | 2.11 | 0.50 | 1.26 | 3.90 | 7.86 |
|  |  | Deccan Peninsula | 8 | 3.38 ± 0.70 | 2.76 | 1.99 | 2.18 | 2.51 | 3.03 | 8.20 |
|  |  | Western Ghats | 10 | 1.95 ± 0.45 | 1.98 | 1.44 | 0.20 | 0.63 | 3.00 | 4.00 |
|  | **Gender** | Male | 11 | 2.77 ± 0.69 | 2.71 | 2.30 | 0.20 | 0.85 | 3.79 | 8.20 |
|  |  | Female | 18 | 2.74 ± 0.41 | 2.70 | 1.76 | 0.20 | 1.96 | 3.54 | 7.86 |
|  | **Ontogeny** | Adult | 24 | 3.32 ± 0.35 | 3.00 | 1.71 | 0.70 | 2.63 | 7.06 | 8.20 |
|  |  | Subadult | 2 | 0.61 ± 0.10 | 0.61 | 0.15 | 0.50 | 0.55 | 0.66 | 0.71 |
|  |  | Neonate | 3 | 0.30 ± 0.10 | 0.20 | 0.17 | 0.20 | 0.20 | 0.35 | 0.50 |
| ***E. c. sochureki*** | **Venom yield** | - | 8 | 50.01 ± 14.38 | 38.54 | 40.69 | 9.92 | 17.90 | 81.98 | 123.20 |

This table summarises venom yield statistics for the ‘big four’ Indian snakes: *N. naja, D. russelii, B. caeruleus,* and *E. c. carinatus* (including its subspecies *E. c. sochureki*). The data are presented as sample size (N), mean ± SEM (standard error of the mean), median, standard deviation (SD), minimum venom yield (min), first quartile (Q1), third quartile (Q3), and maximum venom yield (max). For some samples locality, gender, and/or age were not recorded.
