# Supplementary material for: Hiss and tell: What influences venom yields of India’s big four snakes?
Source: PLoS Negl Trop Dis. 2025 Nov 3;19(11):e0013676. doi: 10.1371/journal.pntd.0013676 (PMC12591399; doi:10.1371/journal.pntd.0013676)
Supplement: Attachment — Submitted filename: PNTD-D-25-01566.pdf [file pntd.0013676.s006.pdf]

PLOS Neglected Tropical Diseases

Hiss and tell: What influences venom yields of India's big four snakes?

--Manuscript Draft--

**Manuscript Number:**	PNTD-D-25-01566
**Full Title:**	Hiss and tell: What influences venom yields of India's big four snakes?
**Short Title:**	What influences venom yields of India's big four snakes?
**Article Type:**	Research Article
**Keywords:**	Venom yield, Naja naja, Daboia russelii, Bungarus caeruleus, Echis carinatus
**Abstract:**	Snake venoms are complex biochemical mixtures that have evolved over millions of years to rapidly subdue prey and deter predators. The potency and amount of venom differ significantly both within and across species, and are largely influenced by their ecology and environment. Understanding venom yields in clinically relevant species is essential for refining treatment approaches for snakebite. To address this knowledge gap, we analysed venom yields from 338 specimens of the “big four” Indian snakes—Naja naja, Daboia russelii, Bungarus caeruleus, and Echis carinatus—collected across diverse biogeographic and agroclimatic zones in India. We systematically compared yields across different genders, regions, and life stages to uncover patterns in venom production and explored the relationship between venom yield and dry weight. Our findings reveal substantial inter- and intraspecific variation in venom yields, with N. naja and D. russelii exhibiting the highest average yields, while B. caeruleus and E. carinatus produced much lower quantities. Geographic variation was significant for B. caeruleus, particularly between the Gangetic Plains and Western Ghats, but not for the other species. No sex-based differences were observed, but life stage was a major determinant, with adults yielding more venom than juveniles or subadults. Comparative analysis with captive populations indicated that captivity may not substantially alter venom yields. By integrating venom yield and toxicity data, we highlight the distinct envenomation strategies among the big four snakes and their implications for snakebites. These insights are crucial for improving antivenom production, clinical management, and understanding the ecological and evolutionary dynamics of Indian snake venoms.
**Additional Information:**	
**Question**	**Response**
**Government Employee**<br><br>Are you or any of the contributing authors an employee of the United States government?<br><br>Manuscripts authored by one or more US Government employees are not copyrighted, but are licensed under a CC0 Public Domain Dedication, which allows unlimited distribution and reuse of the article for any lawful purpose. This is a legal requirement for US Government employees.<br><br>This will be typeset if the manuscript is accepted for publication.	No - No authors are employees of the U.S. government.

Powered by Editorial Manager® and ProduXion Manager® from Aries Systems Corporation

**Financial Disclosure**<br><br>Enter a financial disclosure statement that describes the sources of funding for the work included in this submission. Review the submission guidelines for detailed requirements. View published research articles from *PLOS Neglected Tropical Diseases* for specific examples.<br><br>This statement is required for submission and **will appear in the published article** if the submission is accepted. Please make sure it is accurate.<br><br>**Funded studies**<br>Enter a statement with the following details:<br>• Initials of the authors who received each award<br>• Grant numbers awarded to each author<br>• The full name of each funder<br>• URL of each funder website<br>• Did the sponsors or funders play any role in the study design, data collection and analysis, decision to publish, or preparation of the manuscript?<br><br>Did you receive funding for this work?	Yes
Please add funding details.<br>as follow-up to "**Financial Disclosure**<br><br>Enter a financial disclosure statement that describes the sources of funding for the work included in this submission. Review the submission guidelines for detailed requirements. View published research articles from *PLOS Neglected Tropical Diseases* for specific examples.<br><br>This statement is required for submission and **will appear in the published article** if the submission is accepted. Please make sure it is accurate.	DBT/Wellcome Trust India Alliance Fellowship [IA/I/19/2/504647], Department for International Development (IAVI/BES/KASU/0002), the DST-INSPIRE Faculty Award (DST/INSPIRE/04/2017/000071), and Wellcome Trust grant (223619/Z/21/Z)

**Funded studies**<br>Enter a statement with the following details:<br>• Initials of the authors who received each award<br>• Grant numbers awarded to each author<br>• The full name of each funder<br>• URL of each funder website<br>• Did the sponsors or funders play any role in the study design, data collection and analysis, decision to publish, or preparation of the manuscript?<br><br>Did you receive funding for this work?"	
Please select the country of your main research funder (please select carefully as in some cases this is used in fee calculation).<br>as follow-up to "**Financial Disclosure**<br><br>Enter a financial disclosure statement that describes the sources of funding for the work included in this submission. Review the submission guidelines for detailed requirements. View published research articles from *PLOS Neglected Tropical Diseases* for specific examples.<br><br>This statement is required for submission and **will appear in the published article** if the submission is accepted. Please make sure it is accurate.<br><br>**Funded studies**<br>Enter a statement with the following details:<br>• Initials of the authors who received each award<br>• Grant numbers awarded to each author<br>• The full name of each funder<br>• URL of each funder website<br>• Did the sponsors or funders play any role in the study design, data collection and analysis, decision to publish, or preparation of the manuscript?<br><br>Did you receive funding for this work?"	INDIA - IN
**Competing Interests**	All authors of this manuscript declare that they have no known conflicts of interest

On behalf of all authors, disclose any competing interests that could be perceived to bias this work.<br><br>This statement will be typeset if the manuscript is accepted for publication.<br><br>Review the instructions link below and PLOS NTDs' competing interests policy to determine what information must be disclosed at submission.	related to this publication.
**Data Availability**<br><br>Provide a **Data Availability Statement** in the box below. This statement should detail where the data used in this submission can be accessed. This statement will be typeset if the manuscript is accepted for publication.<br><br>Before publication, authors are required to make all data underlying their findings fully available, without restriction. Review our PLOS Data Policy page for detailed information on this policy. Instructions for writing your Data Availability statement can be accessed via the Instructions link below.	All relevant data are available within the manuscript and its supplementary files.

Manuscript Click here to access/download;Manuscript;Manuscript_v2.pdf

Hiss and tell: What influences venom yields of India's big four snakes?

Prasad Gopalkrishna Gond*, Mihir Kumar*, Ajinkya Unawane*, and Kartik Sunagar@

1. Evolutionary Venomics Lab, Centre for Ecological Sciences, Indian Institute of Science, Bangalore 560012, Karnataka, India.

* These authors contributed equally
@Corresponding author

Abstract

Snake venoms are complex biochemical mixtures that have evolved over millions of years to rapidly subdue prey and deter predators. The potency and amount of venom differ significantly both within and across species, and are largely influenced by their ecology and environment. Understanding venom yields in clinically relevant species is essential for refining treatment approaches for snakebite. To address this knowledge gap, we analysed venom yields from 338 specimens of the “big four” Indian snakes—*Naja naja*, *Daboia russelii*, *Bungarus caeruleus*, and *Echis carinatus*—collected across diverse biogeographic and agroclimatic zones in India. We systematically compared yields across different genders, regions, and life stages to uncover patterns in venom production and explored the relationship between venom yield and dry weight. Our findings reveal substantial inter- and intraspecific variation in venom yields, with *N. naja* and *D. russelii* exhibiting the highest average yields, while *B. caeruleus* and *E. carinatus* produced much lower quantities. Geographic variation was significant for *B. caeruleus*, particularly between the Gangetic Plains and Western Ghats, but not for the other species. No sex-based differences were observed, but life stage was a major determinant, with adults yielding more venom than juveniles or subadults. Comparative analysis with captive populations indicated that captivity may not substantially alter venom yields. By integrating venom yield and toxicity data, we highlight the distinct envenomation strategies among the big four snakes and their implications for snakebites. These insights are crucial for improving antivenom production, clinical management, and understanding the ecological and evolutionary dynamics of Indian snake venoms.

Keywords: Venom yield, *Naja naja, Daboia russelii, Bungarus caeruleus, Echis carinatus*

1. Introduction

India is home to an impressive variety of snakes, with over 426 species recorded to date. Among these, nearly 60 are clinically relevant to humans, requiring clinical intervention to prevent severe symptoms associated with their bites. The so-called "big four" snake species, namely spectacled cobra (*Naja naja),* Russell's viper (*Daboia russelii*)*,* common krait (*Bungarus caeruleus*)*,* and saw-scaled viper (*Echis carinatus*), are said to be responsible for most envenomations and snakebite-related morbidities and fatalities [1].

Snake venoms are intricate biochemical concoctions that have evolved to rapidly incapacitate and/or kill prey [2,3]. The potency and volume of venom produced considerably vary among snakes, and are linked to their ecological roles [4,5]. Understanding venom yields from clinically important snakes is crucial for optimising snakebite treatments, as it provides insight into the volume of venom that can be potentially injected by these snakes into their bite victims.

Senji Laxame also did a similar research in 2019. PLoS Negl Trop Dis 13(12): e0007899. https://doi.org/10.1371/journal.pntd.0007899

To date, only a single study has reported venom yields from three of the four 'big four' snake species in India, focusing exclusively on a captive population in Maharashtra [6]. Although this research observed seasonal variation in venom yields, the controlled conditions of captivity result in an inability to assess the true impact of abiotic factors. No studies to date have generated comprehensive data on venom yields from wild snake populations in India.

We seek to fill this knowledge gap by analysing venom yields from over 338 snake specimens collected from distinct biogeographic/agroclimatic zones across India. We systematically assess differences in venom yields based on gender, geography, and life stage, aiming to identify patterns and underlying variations. The data being reported here are vital for both medical and ecological research.

2. Methodology

2.1. Ethical statement

We have collected snake venom samples with the required permissions from the following states: Andhra Pradesh (Permit No: 16284/2016/WL-3, 07.02.2022), Goa (Permit No: 2-66-WL-RESEARCH PERMISSIONS-FD-2022-23-Vol.IV/858, 27.05.2022), Karnataka (Permit No: PCCF(WL)/E2/CR-06/2018-19, 23.02.2022), Kerala (Permit No: KFDHQ-1006/2021-CWW/WL10, 10.06.2022), Maharashtra Desk-22/(8)/WL/Research/CR-60(17-18)/3349/21-22, 21/03/2022), Madhya Pradesh (No./M.CH-II/Research/F-214/21932), Rajasthan (Permit No: F19(29) Permission/ CWLC/ 2017-18/ 595, 09/05/2018), Tamilnadu (WL5(A)/33005/2017, Permission No. 61/2023), Uttarakhand (Letter No: 2519/5-6, 21.03.2024), and West Bengal (Memo No:1023/WL/4R-12/2022, 21.04.2022) India. No animal or human samples were utilised in this study, and the LD_{50} values have been reported from our previous publications (Supplementary Table 1).

2.2. Venom extraction and storage

Between 2021 and 2024, snake venom samples were collected from wild-caught snakes during rescue operations coordinated by the State Forest Department and local snake rescuers. After extracting the venom, the snakes were released back into their natural habitat under the forest department's supervision. Each snake was identified to the species level, ensuring that only healthy individuals were chosen for venom extraction.

Experienced herpetologists employed safe handling methods, using acrylic restraining tubes to manually restrain them. Venom was collected by encouraging the snake to bite onto a sterile parafilm stretched over a beaker, without applying external pressure to the venom gland (Supplementary Figure 1). The extracted crude venom was promptly measured and placed in a vapour shipper for transportation. Following lyophilisation, the venom samples were stored at –80 °C until ready for further processing. The dry weight of the lyophilised venom samples was measured with a high-precision microbalance (Sartorius AG, Göttingen, Germany).

2.3. Statistical analysis

All statistical analyses were conducted using GraphPad Prism (version 10.0, GraphPad Software, USA; www.graphpad.com). Initially, venom yield data underwent a normality check with the Shapiro-Wilk test [7]. Since the data did not follow a normal distribution, non-parametric tests were utilised. To compare venom yields among multiple groups, the Kruskal-Wallis test was applied [8], followed by Dunn’s post hoc test with Benjamini-Hochberg adjustment for multiple-comparison corrections [9]. Effect sizes for the Kruskal-Wallis tests were determined using epsilon-square (R4.5.1; www.R-project.org). Comparisons between two groups, the Mann-Whitney U test was used [10], followed by a suitable post hoc test, with effect sizes calculated using rank-biserial correlation (r) [11].

3. Results

3.1 Venom yields from the pan-Indian populations of the 'big four' snakes

A total of 338 venom samples were collected across various biogeographic regions in India, including the coastal plains, Deccan Plateau, Gangetic Plains, semi-arid regions, Western Ghats and the deserts (Table 1 and Figure 1).

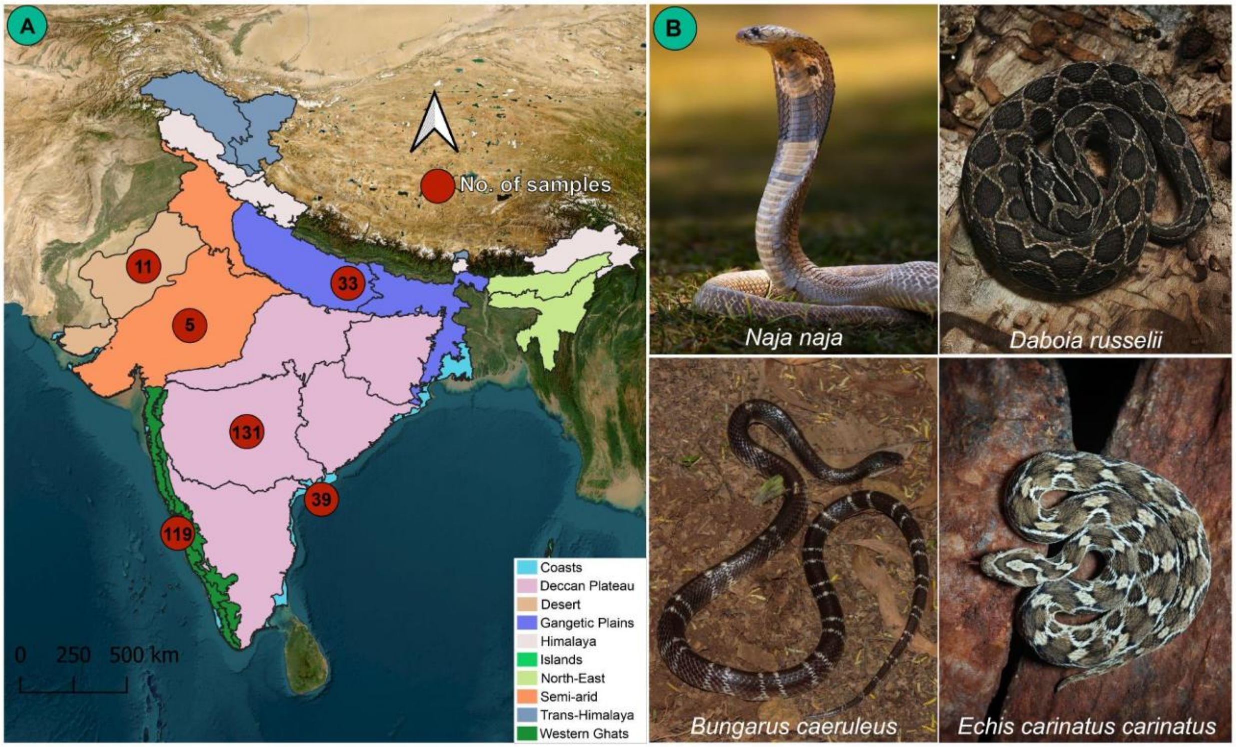

Figure 1. (A) The map illustrates the geographic distribution of venom samples collected throughout India. Sampling efforts are depicted within the six major Biogeographic Zones. The map of India shown in panel (A) was prepared using QGIS 3.40. Red circles accompanied by numbers denote the total number of venom samples collected from each region. **(B)** Representative images of the 'big four' snakes analysed in this work are shown: *N. naja*, *D. russelii*, *B. caeruleus*, and *E. c. carinatus*.

Across all species and regions (N = 338 milking events), venom yields measured as dry weight range from 0.1 mg to 800.4 mg per milking event, with a median yield of 58 mg (Table 2). The mean yield for *N. naja* was 136 mg (0.5–800.4 mg), for *D. russelii* 107 mg (0.5–749 mg), for *B. caeruleus* 9.0 mg (0.1–32 mg), and for *E. c. carinatus* 2.8 mg (0.2–8.2 mg). *E. c. carinatus* yielded the lowest average venom, significantly less than the larger elapid and viperid species ($p<0.0001$), *N. naja* and *D. russelii*, while *B. caeruleus* yielded intermediate amounts with considerable variation ($p<0.0001$).

Species	Biogeographic zone						Total
	Coast	Deccan Plateau	Gangetic Plains	Semi-arid	Western Ghats	Desert	
B. caeruleus	3	14	8	2	16	NA	43
N. naja	21	55	11	3	51	3	144
D. russelii	3	54	14	NA	42	NA	113
E. c. carinatus	12	8	NA	NA	10	NA	30
E. c. sochureki	NA	NA	NA	NA	NA	8	8
**Total**	39	131	33	5	119	11	338

Table 1. Venom samples from a number of specimens of medically important snakes were obtained across six major biogeographic zones of India. Species include *B. caeruleus*, *N. naja*, *D. russelii*, *E. c. carinatus*, and *E. c. sochureki*. NA: not available.

Venom yields showed considerable variation among the ‘big four’ species and the *E. carinatus* subspecies investigated (Table 1, Figure 2A and Supplementary Table 2). *B. caeruleus* and *E. c. carinatus* had the lowest mean yields, with *B. caeruleus* averaging 9.0 ± 1.28 mg (range: 0.1–32 mg) and *E. c. carinatus* averaging 2.8 ± 0.35 mg (range: 0.2–8.2 mg). *E. c. sochureki* yielded intermediate amounts, with a mean of 50.8 ± 14.4 mg (range: 9.9–123 mg). The highest mean yields were recorded for *D. russelii* (107 ± 9.61 mg, range: 0.5–749 mg) and *N. naja* (136 ± 11.2 mg, range: 0.5–800 mg).

Species	N	Mean ± SEM	Median	SD
N. naja	144	136 ± 11.2	83.6	134
D. russelii	115	107 ± 9.61	86.1	103
B. caeruleus	41	9.0 ± 1.28	6	8.18
E. c. carinatus	30	2.76 ± 0.35	2.76	1.9
E. c. sochureki	8	50 ± 14.4	38.5	40.7

Above the authors indicated 50.8

Table 2. Descriptive statistics of dry venom yields (mg) for four snake species and one subspecies sample. N = Number of snakes, SD = Standard Deviation, SEM = Standard Error of the Mean.

The ranking of median venom yields mirrored the means, with *E. c. carinatus* producing the lowest median yield (2.76 mg) and *D. russelii* the highest (86.1 mg) (Figure 2A). Notably, the standard deviations for *N. naja* (SD = 134.0 mg) and *D. russelii* (SD = 103 mg) were substantial, indicating significant intraspecific variability and the presence of both low- and high-yielding individuals within these species.

The SD by itself cannot explain intraspecific variations.

Venom yield differed markedly between species, with *N. naja* and *D. russelii* producing significantly higher amounts than *B. caeruleus* and *E. carinatus* (Kruskal–Wallis χ^2 = 139.6, df = 4, $p < 0.0001$; Figure 2A). Geographic comparisons revealed notable differences between zones for *D. russelii* (Figure 4A) and *B. caeruleus* (Figure 5A), while *N. naja* and *E. carinatus* did not exhibit such spatial variation (Figure 3A and Figure 6A). Sex-specific differences were not found across any species under investigation (Figure 2C). Ontogenetic assessment showed that venom yield increases substantially from juvenile to adult stages ($p<0.05$), highlighting life stage as a key factor influencing venom production (Figure 2D).

Where are figures 2B, 2C, 2D, 3A, 3B and 3C mention?

Where are figures 4B, and 4C mention?

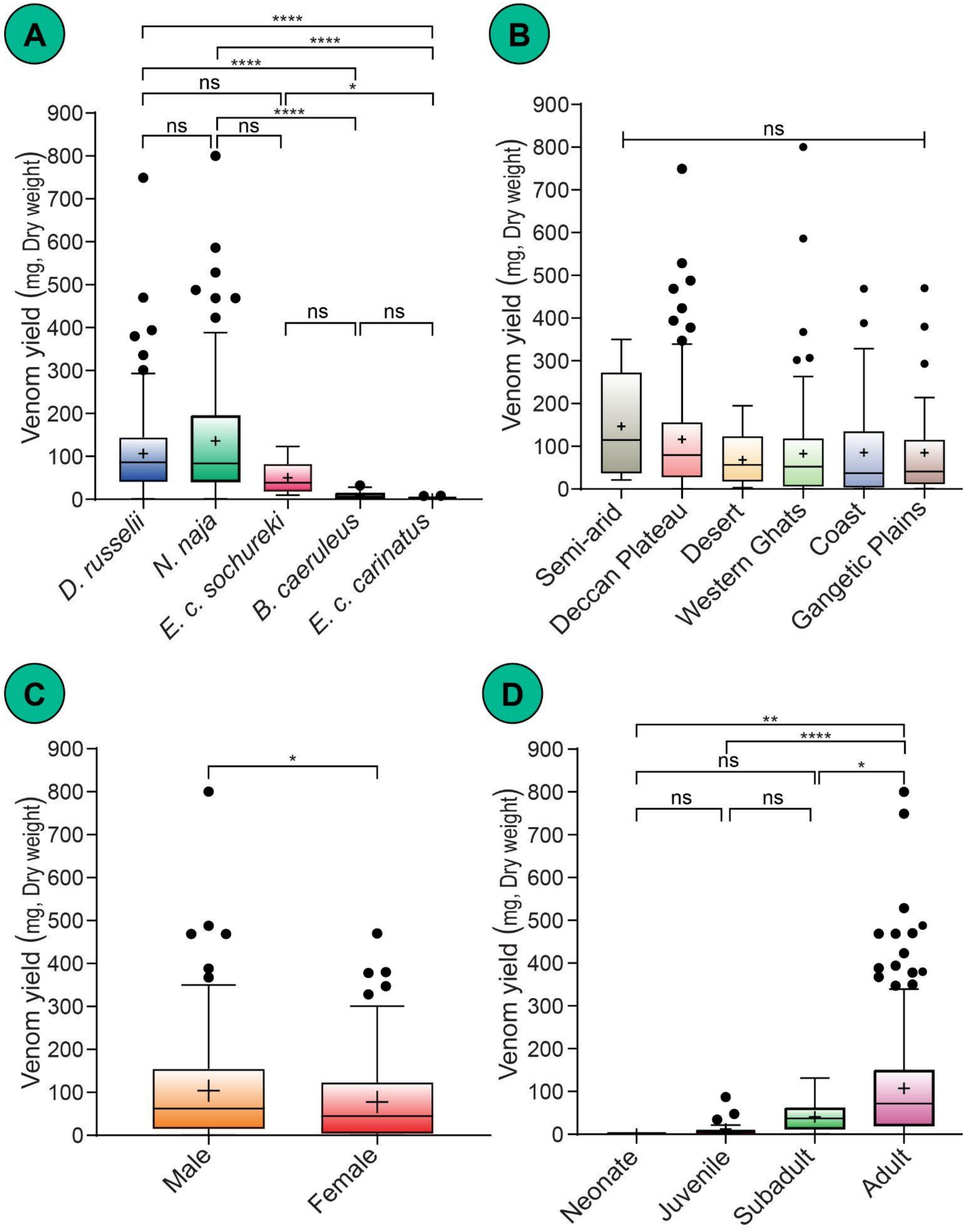

Figure 2. Patterns of venom yield in the 'big four' snakes: (A) among species, (B) among biogeographic regions, (C) between the two sexes, and (D) throughout ontogenetic

stages. Data are represented as Tukey boxplots showing median, interquartile ranges, and outliers (dots). Statistical comparisons were performed using non-parametric tests. Here, *: p<0.05; **: p<0.01; ****: p<0.0001; ns: non-significant; +: mean.

3.1.1 ***Naja naja***

Venom yield in *N. naja* (n = 144; mean = 136 mg, median = 83.6 mg, range: 0.5–800.4 mg) did not differ significantly across the six biogeographic zones of India (Kruskal–Wallis $\chi^2 = 3.103$, df = 5, p = 0.684; Figure 3A). Although the highest median yield was observed in the semi-arid zone (194 mg) and the lowest in the Western Ghats (73 mg), these differences were not statistically significant (p=0.68). Comparisons between sexes revealed no significant variation in venom yield (Mann-Whitney U = 858.5, p = 0.254; Figure 3B). Males had a mean venom yield of 141 mg, while females averaged 114 mg, with considerable overlap in their distributions. However, analysis by life stage showed statistically significant differences (Kruskal–Wallis $\chi^2 = 39.804$, df = 2, p < 0.0001). Adults produced the highest median venom yield at 125 mg, whereas subadults and juveniles had much lower median yields of 47.6 mg and 6.5 mg, respectively (Figure 3C and Supplementary Table 2).

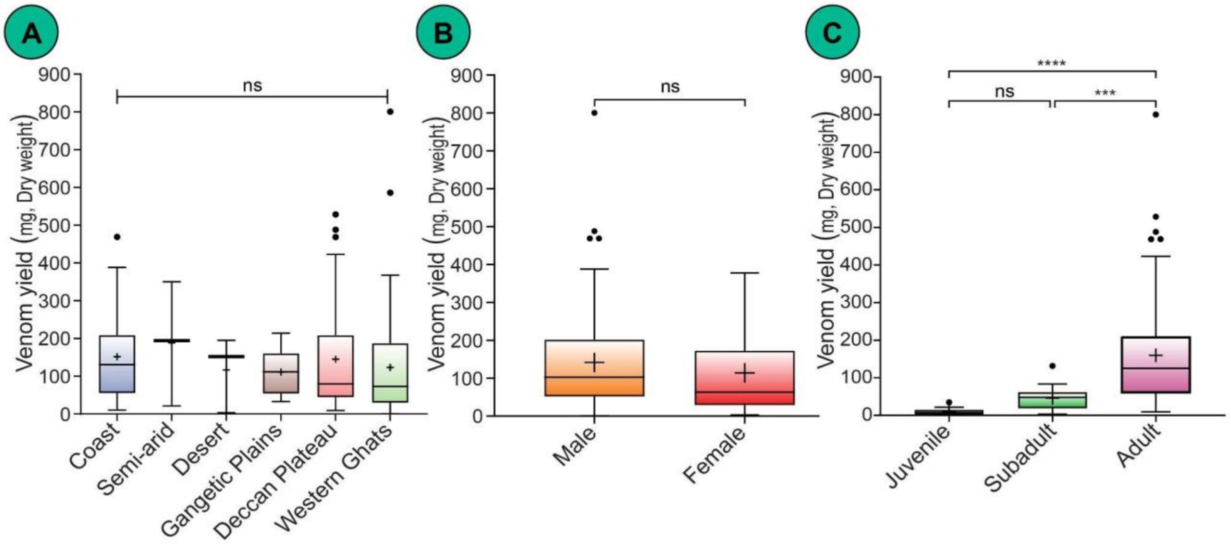

Figure 3. Venom yield in *N. naja*: (A) among biogeographic regions, (B) between the two sexes, and (C) throughout ontogenetic stages. Data are represented as Tukey boxplots showing median, interquartile ranges, and outliers (dots). Statistical comparisons were performed using non-parametric tests. Here, ***: p<0.001; ****:p<0.0001; ns: non-significant; +: mean.

3.1.2 ***Daboia russelii***

Venom yield in *D. russelii* (n = 115; mean = 107 mg, median = 86.1 mg, range: 0.5–749 mg) varied significantly across India's biogeographic zones (Kruskal–Wallis $\chi^2 = 13.05$, df = 4, p = 0.011; Figure 4A), but post hoc Dunn's tests with multiple comparison correction did not find significant pairwise differences (adjusted p>0.05). The highest median yield was recorded in the Deccan Plateau (126 mg), while the Gangetic Plains

had the lowest (28 mg). There were no significant differences in venom yield between male and female *D. russelii* (Mann-Whitney U = 727.5, p = 0.383; Figure 4B), with females (n = 48, mean = 108 mg, median = 102 mg) and males (n = 27, mean = 85.3 mg, median = 70 mg) showing considerable overlap in their distributions. Analysis by life stage indicated significant differences in venom yield (Kruskal–Wallis $\chi^2 = 27.777$, df = 2, $p < 0.001$). Adults had the highest median yield at 95.8 mg, subadults showed intermediate yields (median = 33.2 mg), and juveniles had the lowest yields (median = 3 mg; Figure 4C and Supplementary Table 2).

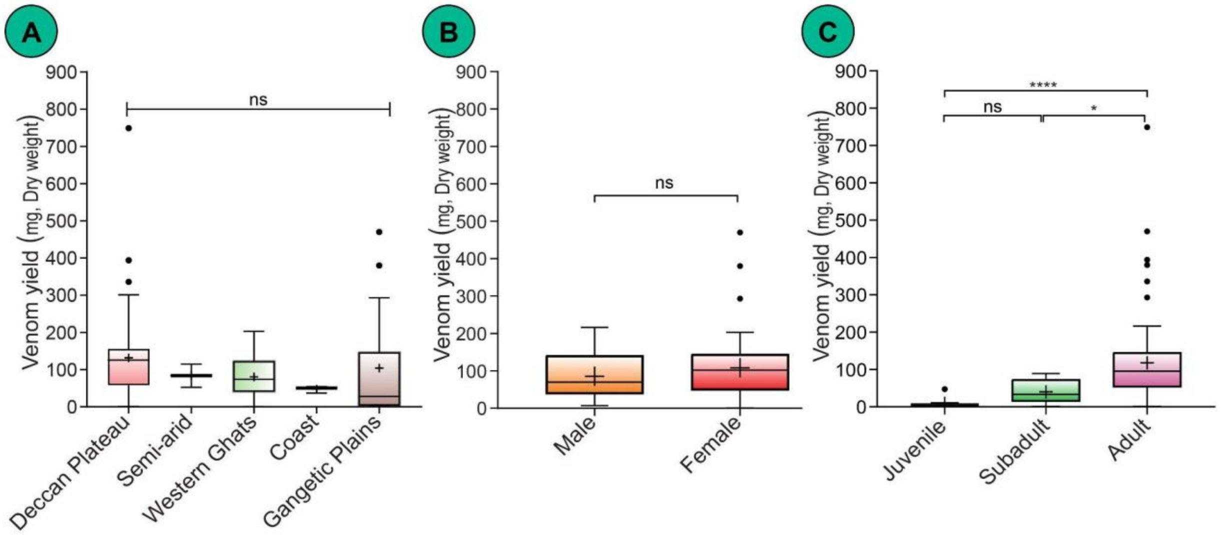

Figure 4. Patterns of venom yield in *D. russelii*: (A) total yield, (B) variation among biogeographic regions, (C) sex differences, and (D) variation throughout ontogenetic stages. Data are represented as Tukey boxplots showing median, interquartile ranges, and outliers (dots). Statistical comparisons were performed using non-parametric tests. Here, *: p<0.05; ****: p<0.0001; ns: non-significant; +: mean.

3.1.3 *Bungarus caeruleus*

Venom yield in *B. caeruleus* (n = 41; mean = 9.0 mg, median = 6 mg, range: 0.1–32 mg) showed significant geographic variation (Kruskal–Wallis $\chi^2 = 7.824$, df = 3, p = 0.0498; Figure 5A). Dunn's post-hoc analysis identified significant differences between the Gangetic Plains and Western Ghats (Adjusted p = 0.0051), with the highest median yield observed in the Gangetic Plains (14.6 mg) and the lowest in the Western Ghats (4 mg). Sex-based analysis revealed no statistically significant differences in venom yield between the males and females (Mann-Whitney U = 75, p = 0.479; Figure 5B), as male (mean = 9.62 mg, median = 6.5 mg) and female (mean = 8.62 mg, median = 5.13 mg) yields overlapped substantially (Supplementary Table 2). Ontogenetic analysis was not conducted for *B. caeruleus* due to the limited number of venom samples from the juvenile and subadult specimens, though adequate sample sizes were available for *N. naja* and *D. russelii*.

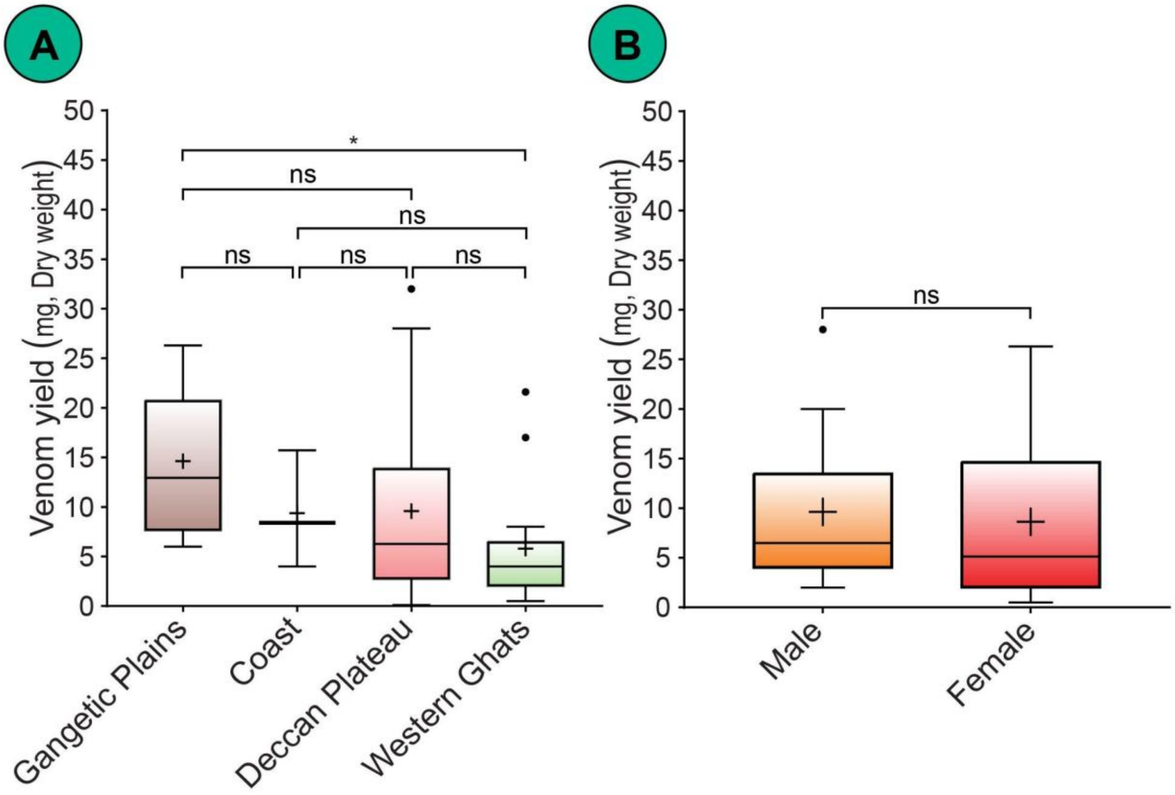

Figure 5. Venom yield in *B. caeruleus*: (A) by biogeographic region and (B) by sex. Data are represented as Tukey boxplots showing median, interquartile ranges, and outliers (dots). Statistical comparisons were performed using non-parametric tests. Here, *: $p<0.05$; ns: non-significant: +: mean.

3.1.4 *Echis carinatus carinatus*

Venom yield in *E. c. carinatus* ($n = 30$; mean = 2.76 mg, median = 2.76 mg, range: 0.2–8.2 mg) did not vary significantly across the Coast, Deccan Plateau, and Western Ghats biogeographic zones (Kruskal–Wallis $\chi^2 = 2.175$, $df = 2$, $p = 0.337$; Figure 6A and Supplementary Tables 2), with median yields consistently ranging from 1.98 to 3.46 mg. Sex-based analysis showed no significant differences in venom yield (Mann-Whitney $U = 97$, $p = 0.946$; Figure 6B), as males (mean = 2.77 mg, median = 2.71 mg) and females (mean = 2.74 mg, median = 2.71 mg) had nearly identical values (Supplementary Table 2). However, ontogenetic comparisons revealed highly significant differences (Kruskal–Wallis $\chi^2 = 11.72$, $df = 3$, $p < 0.0028$). Adults produced the highest median venom yield at 3 mg, while subadults and neonates had substantially lower yields, with medians below 1 mg (Figure 6C and Supplementary Table 2).

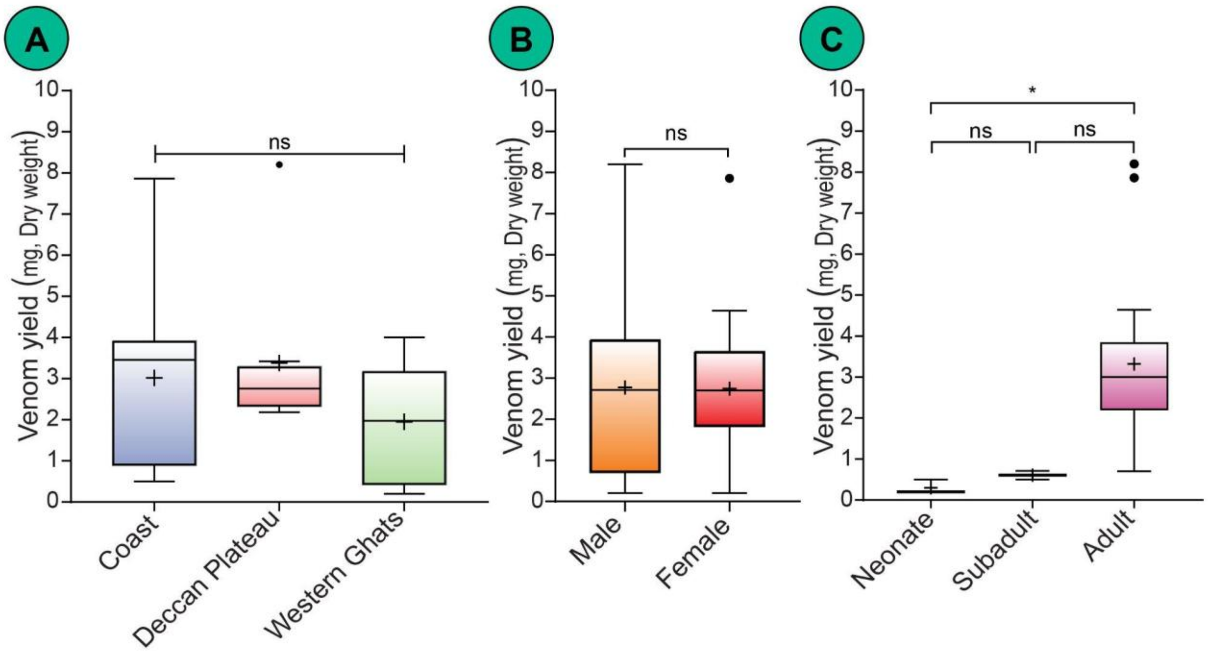

Figure 6. Venom yield in *E. c. carinatus*: (A) by biogeographic regions, (B) by sex, and (C) by ontogenetic stage. Data are represented as Tukey boxplots showing median, interquartile ranges, and outliers (dots). Statistical comparisons were performed using non-parametric tests. Here, *: p<0.05; ns: non-significant; +: mean.

4. Discussion

Our comprehensive and biogeographically inclusive assessment of venom yield in the pan-Indian populations of 'big four' snakes provides the first detailed insights into how ontogeny, sex, and geography affect venom production in wild populations of snakes. Snakes were captured from the wild for sample collection and then safely released back at their original locations. This approach proved highly effective and non-harmful, as demonstrated by the recapture of a previously marked *E. carinatus* individual in the same area in subsequent years.

Our analyses revealed that venom yields varied significantly among species, with *N. naja* (mean = 144 ± 1.2 mg) and *D. russelii* (mean = 115 ± 9.61 mg) producing the largest quantities, while *B. caeruleus* (mean = 8.95 ±1.28 mg) and *E. carinatus* (mean = 2.76 ± 0.35 mg) yielded much less (Table 1). This pattern likely reflects the influence of body size, as larger snakes had higher venom yields than smaller species—a trend supported by previous research showing a positive correlation between body size and venom yield [12–15].

There is not measures of body sizes in the reseach, how can the authors state this with no data support?

Biogeographic variation in venom yield was notable for *B. caeruleus,* with individuals from the Gangetic Plains producing substantially higher venom yields compared to those from the Western Ghats (Adjusted p = 0.0051; Figure 5A). In contrast, *N. naja, D. russelii*, and

E. c. carinatus did not exhibit significant biogeographic differences in venom yield (Figures 3A, 4A, and 6A). Interestingly, sexual dimorphism did not seem to influence the venom yield either, which contrasts with some earlier studies that reported higher yields in females [12]. The distributions of venom yield for males and females of all four species overlapped considerably (Supplementary Table 2).

Ontogenetic shifts in venom compositions, functions and potencies have been reported for *D. russelii* in India [16], while these shifts remain unstudied for the other three members of the 'big four' snakes. Consistent with these findings, ontogenetic analysis demonstrated that life stage is a primary determinant of venom output. Adults produced the highest median yields, while subadults and juveniles yielded much less (Supplementary Table 2). For example, adult *N. naja* had a median yield of 125 mg, compared to 47.6 mg for subadults and just 6.5 mg for juveniles. Similar trends were observed in *D. russelii* and *E. c. carinatus* (Supplementary Table 2). Ontogenetic analysis was not performed for *B. caeruleus* because there were too few venom samples from juvenile and adult individuals to allow meaningful comparison. Collecting krait venoms from wild populations is challenging due to their elusive nature [17].

We also compared venom yields from wild-caught snakes to previously published data from captive populations. No significant differences were found between wild and captive yields (Supplementary Figure 2), suggesting that captivity may not substantially alter venom yields under artificial conditions. However, previous research has shown that the first milking session in captivity often yields more venom than subsequent extractions, and that factors, such as health, feeding, and extraction intervals, can influence output [18]. The only way to arrive at a conclusive answer to this question is by measuring venom yields before and after the snakes are housed in captivity.

The methodology section does not mention how this resutls or values were obtained.

Where all these data comes from? There is not mention of bibliography.

LD_{50} values and venom yield data were analysed together to illustrate the distinct envenomation strategies of the big four snakes. *B. caeruleus* stands out for its exceptional potency (LD_{50} = 0.11 mg/kg) despite having the lowest average venom yield (9 mg). In contrast, *N. naja* possessed moderate toxicity (LD_{50} = 0.42 mg/kg) with the highest average venom yield (136 mg), which likely contributes to the higher rates of snakebite mortality caused by this species. Similarly, *D. russelii* produced very high venom yields (107 mg) and a comparatively low LD_{50} (0.31 mg/kg). On the other hand, *E. c. carinatus* has the least toxic venom (LD_{50} = 0.64 mg/kg) and the smallest venom yield (2.8 mg), yet remains a leading cause of envenomation. These findings indicate that snakebite burden is shaped not just by venom potency alone, but by the interplay of potency, venom yield and other factors (Supplementary Tables 1 and 3).

Overall, this research fills a critical gap in the understanding of venom yield variation in Indian snakes, highlighting the importance of size, geography, and life stage, while finding no evidence for the influence of sexual dimorphism. These findings have important

implications for antivenom production, clinical management of snakebite, and ecological and venomics studies. By providing robust, regionally representative data, the study supports efforts to improve snakebite treatment and deepen knowledge of venom biology in India.

Acknowledgements

This work was supported by the DBT/Wellcome Trust India Alliance Fellowship [IA/I/19/2/504647] awarded to KS. This work was also supported by the Department for International Development (IAVI/BES/KASU/0002), the DST-INSPIRE Faculty Award (DST/INSPIRE/04/2017/000071), and Wellcome Trust grant (223619/Z/21/Z) to KS. Venom sampling equipment and expeditions were supported by USV Private Limited (http://www.usvindia.com). The authors are sincerely thankful to the following State Forest Departments for the kind support and permits for venom collection: Andhra Pradesh, Goa, Karnaka, Kerala, Maharashtra, Madhya Pradesh, Rajasthan, Tamil Nadu, Uttarakhand and West Bengal. The authors are thankful to Ajay Kartik and Gerry Martin for their invaluable assistance in collecting samples. The authors are thankful to Akash Verma (IFS, Uttarakhand FD), Mr. Anwar Yusuf (ACF, Kerala FD), Dr. Siddharth Bhatia, and Gnaneshwar CH for their support during venom collection. We would like to thank the following snake rescuers for assistance with sample collection: Vivek Sharma, Amrit Singh, Charan Desai, Mirjoy Mathew, Abhijeet Patil, Swanand Patil; Sachin Gowda, Anand Chitti, Shivani Chitti, Susmita Nesarikar, Jeet Roche, Bhuwan Devadiga, Afsar Hussain, Aditya Vattam, Amit Tapase, Kushal Kori, Ajith, Omkar Pai, Vinayak Bhatt, Suhas Hegade, Pavan Naik, Afran, Poorna, Akshay Sheth, Sammilan Shetty; Javad Kudukkan; Vivek Rathod, Kiran Kumar, and Sabbavarapu Madhu; Shankar Wadkar, Pratik Mahamuni, Deepak Pacharne; Vishwa; Vishal Varma, Shyam Govindsar, and Padma Singh Rathore; Sudarshan Singh, and Arshad Khan; and Suvrajyoti Chatterjee, Nandu Kumar. We also thank others who contributed but are not listed here.

Author contributions

Conceptualisation: KS; Investigation: PGG, MK, AU and KS; Data curation: PGG and AU; Methodology: PGG, AU and MK; Resources: KS; Formal analysis: MK, PGG, AU and KS; Interpretation: PGG, MK, AU and KS; Visualisation: PGG, MK and KS; Writing - original draft: KS, PGG, MK and AU; Writing - review & editing: PGG, MK, AU and KS; Supervision: KS; Funding acquisition: KS.

Conflict of interest

All authors of this manuscript declare that they have no known conflicts of interest related to this publication.

References

1. Mohapatra B, Warrell DA, Suraweera W, Bhatia P, Dhingra N, Jotkar RM, et al. Snakebite mortality in India: a nationally representative mortality survey. PLoS Negl Trop Dis. 2011;5: e1018. doi:10.1371/journal.pntd.0001018

2. Sunagar K, Morgenstern D, Reitzel AM, Moran Y. Ecological venomics: How genomics, transcriptomics and proteomics can shed new light on the ecology and evolution of venom. J Proteomics. 2016;135: 62–72. doi:10.1016/j.jprot.2015.09.015

3. Casewell NR, Jackson TNW, Laustsen AH, Sunagar K. Causes and consequences of snake venom variation. Trends Pharmacol Sci. 2020;41: 570–581. doi:10.1016/j.tips.2020.05.006

4. Healy K, Carbone C, Jackson AL. Snake venom potency and yield are associated with prey-evolution, predator metabolism and habitat structure. Ecol Lett. 2019;22: 527–537. doi:10.1111/ele.13216

5. Sarangi N, Laxme RRS, Sunagar K. Significant serpents: Predictive modelling of bioclimatic venom variation in Russell's viper. PLoS Negl Trop Dis. 2025;19: e0012949. doi:10.1371/journal.pntd.0012949

6. Tumbare S, Khadilkar M. Venom yield of major Indian poisonous Snakes in captivity. J Bombay Vet Coll. 2004.

7. Shapiro SS, Wilk MB. An analysis of variance test for normality (complete samples). Biometrika. 1965;52: 591–611. doi:10.1093/biomet/52.3-4.591

8. Kruskal WH, Wallis WA. Use of ranks in one-criterion variance analysis. J Am Stat Assoc. 1952;47: 583. doi:10.2307/2280779

9. Dunn OJ. Multiple comparisons using rank sums. Technometrics. 1964;6: 241. doi:10.2307/1266041

10. Mann HB, Whitney DR. On a test of whether one of two random variables is stochastically larger than the other. The annals of mathematical statistics. 1947;

50–60.

11. Ben-Shachar M, Lüdecke D, Makowski D. Effectsize: Estimation of effect size indices and standardized parameters. J Open Source Softw. 2020;5: 2815. doi:10.21105/joss.02815

12. Braga JRM, Oitaven LPC, Da Rocha MMT, Vieira SEM, Rúbio DT, Sant'anna SS, et al. Influence of size, sex and age on venom yield of Bothrops leucurus (Serpentes, Viperidae) in captivity conditions. Basic Appl Herpetol. 2022;36: 31–45. doi:10.11160/bah.233

13. Dissanayake DS, Rajapakse RPVJ, Kularatne SAM. An evaluation on factors influencing venom yield in spectacled cobra (Naja naja). Sri Lanka; 2015.

14. Mirtschin PJ, Shine R, Nias TJ, Dunstan NL, Hough BJ, Mirtschin M. Influences on venom yield in Australian tigersnakes (Notechis scutatus) and brownsnakes (Pseudonaja textilis: Elapidae, Serpentes). Toxicon. 2002;40: 1581–1592. doi:10.1016/s0041-0101(02)00175-7

15. de Roodt AR, Dolab JA, Galarce PP, Gould E, Litwin S, Dokmetjian JC, et al. A study on the venom yield of venomous snake species from Argentina. Toxicon. 1998;36: 1949–1957. doi:10.1016/s0041-0101(98)00119-6

16. Senji Laxme RR, Khochare S, Bhatia S, Martin G, Sunagar K. From birth to bite: the evolutionary ecology of India's medically most important snake venoms. BMC Biol. 2024;22: 161. doi:10.1186/s12915-024-01960-8

17. Rashmi U, Bhatia S, Nayak M, Khochare S, Sunagar K. Elusive elapids: biogeographic venom variation in Indian kraits and its repercussion on snakebite therapy. Front Pharmacol. 2024;15: 1443073. doi:10.3389/fphar.2024.1443073

18. Minton SA Jr. Variation in Yield and Toxicity of Venom from a Rattlesnake (Crotalus atrox). Copeia. 1957;1957: 265. doi:10.2307/1439150

Click here to access/download
Supporting Information
Supplementary Figures_v2.pdf

Supplementary Tables

Click here to access/download
Supporting Information
Supplementary Tables_v2.pdf
