# Supplementary material for: Hiss and tell: What influences venom yields of India’s big four snakes?
Source: PLoS Negl Trop Dis. 2025 Nov 3;19(11):e0013676. doi: 10.1371/journal.pntd.0013676 (PMC12591399; doi:10.1371/journal.pntd.0013676)
Supplement: Attachment — Submitted filename: PNTD-D-25-01566_reviewer comments.docx [file pntd.0013676.s005.docx]

**Overall Assessment**

This manuscript presents a pan-India analysis of venom yields from wild “big four” snakes (Naja naja, Daboia russelii, Bungarus caeruleus, Echis carinatus), spanning six biogeographic zones and 338 milking events. It documents inter/intraspecific and ontogenetic differences and contrasts wild yields with captive reports. The dataset is valuable and clinically relevant.

**Major Strengths**

1. Large scale and ecological validity: The collection of 338 venom samples from wild populations across multiple biogeographic zones is a great effort. This provides data that is more ecologically relevant and representative of actual snakebite scenarios than previous studies reliant on captive snakes.
2. Methodology: The use of non-parametric statistics is appropriate for the non-normally distributed data.
3. Clinically critical insights: The integration of venom yield data with known LD50 values to discuss envenomation strategies (e.g., high yield/moderate toxicity of *N. naja* vs. low yield/extreme potency of *B. caeruleus*) is a standout contribution. This directly informs risk assessment and it is important for snakebite treatment

**Recommendations**

1. **Introduction:**

The introduction is concise and effective. The early statements in the first paragraph should be referenced

2. **Materials and methods**

**Seasonality and environmental covariates:** In this study, years of collection (2021–2024) are available but not analyzed. Adding month/season to the model would visualize seasonal yield distributions by species. This could reveal phenology-linked dynamics of venom yield.

3. **Results**

Statistical reporting for the Kruskal-Wallis test on *D. russelli* geographic variation (p=0.011), the post-hoc test found no significant pairs. This should be briefly discussed in the results text as it suggests that while overall variation exists, specific regional pairwise differences are not stark enough to show significance at multiple-testing correction.

4. **Discussion**
1. Our comprehensive and biogeographically inclusive assessment of venom yield in the pan-Indian populations of ‘big four’ snakes provides the first detailed insights into how ontogeny, sex, and geography affect *venom production* in wild populations of snakes.

**Recast to read-**

Our comprehensive and biogeographically inclusive assessment of venom yield in the pan-Indian populations of ‘big four’ snakes provides the first detailed insights into how ontogeny, sex, and geography affect *venom yield* in wild populations of snakes.

2. Adults produced the highest median yields, while subadults and juveniles yielded much less (Supplementary Table 2). For example, adult N. naja had a median yield of 125 mg, compared to 47.6 mg for subadults and just 6.5 mg for juveniles. – *It is appropriate to compare the mean data in this instance rather than the median data*
3. LD₅₀ values and venom yield data were analysed together to illustrate the distinct envenomation strategies of the big four snakes. B. caeruleus stands out for its exceptional potency (LD₅₀ = 0.11 mg/kg) despite having the lowest average venom yield (9 mg). *– The statement is misleading because from the results, E. c. carinatus had the lowest venom yield and not B. caeruleus*
4. Overall, this research fills a critical gap in the understanding of venom yield variation in Indian snakes, highlighting the importance of size, geography, and life stage, while finding no evidence for the influence of *sexual dimorphism*.

**Recast to read-**

Overall, this research fills a critical gap in the understanding of venom yield variation in Indian snakes, highlighting the importance of size, geography, and life stage, *while finding no* evidence *of sex-related differences*

**Conclusion**

This manuscript represents a landmark study in clinical toxinology. The data is robust, the analysis is sound, and the conclusions are well-supported and highly significant. The authors have provided a vital resource for improving snakebite management in India. The minor suggestions above are intended to further polish an already good manuscript.
