# Supplementary material for: Hiss and tell: What influences venom yields of India’s big four snakes?
Source: PLoS Negl Trop Dis. 2025 Nov 3;19(11):e0013676. doi: 10.1371/journal.pntd.0013676 (PMC12591399; doi:10.1371/journal.pntd.0013676)
Supplement: Attachment — Submitted filename: Reviewer comments_Gond et al. 2025.docx [file pntd.0013676.s007.docx]

This study represents a major piece of work, with over 338 venom samples collected across India being used to investigate venom yields of India’s big four snakes. I commend the authors for such an extensive and important study. Venom yield is often overlooked in toxinology studies; thus, I believe this work is of high value to the scientific community, hopefully encouraging toxinologists to incorporate venom yields into their analyses.

Based off the novelty, extensive data collection, and applicability to future research, I recommend this research paper be accepted for publication pending major revisions.

**Major:**

**Introduction**

**Point 1:** Although I commend the authors for having a concise introduction, it is too short. The introduction lacks impact and detail. I would like to see more literature on the activity of these snakes’ venoms across the biogeographic regions investigated in the study. What is the activity? How does it vary across regions? And most importantly, considering this variation why is studying venom yield important for these snakes? Why might you expect yields to differ across biogeographic regions? Diet?

**Methods**

**Point 2:** “2.3 Statistical analysis”. A Supplementary material file of the statistical assumptions (e.g. normality tests) and outputs should be included. Additionally, another supplementary file containing all raw data should be provided, as well as the R scripts used to test effect sizes. Providing this will make the studies analyses completely transparent and reproducible.

**Discussion**

**Point 3:** Paragraph 6: “These findings indicate that snakebite burden is shaped not just by venom potency alone, but by the interplay of potency, venom yield and other factors (Supplementary Tables 1 and 3).” This is a major finding and should not just be reserved for supplementary material. A figure and/or table showing the comparison between venom yield and LD50 should appear in the results section.

**Minor:**

**Introduction**

**Point 1:** The sentences: “India is home to an impressive variety of snakes, with over 426 species recorded to date. Among these, nearly 60 are clinically relevant to humans, requiring clinical intervention to prevent severe symptoms associated with their bites.”, needs a reference. Consider citing <https://reptile-database.reptarium.cz/>.

**Methods**

**Point 2:** “2.1 Ethical Statement”. This can be changed to “2.1 Snake venom samples”. Expand paragraph to include information on what species’ venom was collected and from where (biogeographic zone). Also include how many venom samples were collected (A total of 338). I know this information is in the authors’ supplementary data, but it fits better in the main paper. After reading this section, the reader should know the following main points: 338 individual snakes were milked encompassing four species, including male and female snakes; neonate, juvenile, subadult, and adult snakes, and snakes from different biogeographic regions.

**Point 3:** Animal samples (venom) were used in the study, thus reword the following sentence: “No animal or human samples were utilised in this study, and the LD50 values have been reported from our previous publications (Supplementary Table 1)”.

**Results**

**Point 4:** In Figure 1B. the photo credits are missing. Please provide.

**Point 5:** The paragraph starting with “Across all species and regions (N = 338 milking events),………..” should appear after Table 1.

**Point 6:** The sentence: “Sex-specific differences were not found across any species under investigation (Figure 2C).”, needs rewording. Figure 2C shows a significant difference between males and females. I recommend the authors make it clear that although there is a significant difference overall between males and females, this difference was not observed within species.

**Point 7:** The phrase “showing considerable overlap in their distributions.” is used throughout the methods to describe overlap of the data points between groups. Since differences in biogeographic regions are explored, the use of “distributions” may lead to confusion (distributions sounds like it is related to geography). Consider rewording.

**Discussion**

**Point 8:** The sentences: “Snakes were captured from the wild for sample collection and then safely released back at their original locations. This approach proved highly effective and non-harmful, as demonstrated by the recapture of a previously marked *E. carinatus* individual in the same area in subsequent years.” Would fit better in the methods section. Additionally, a reference is needed.

**Point 9:** In discussion (second paragraph) “while *B. caeruleus* (mean = 8.95 ±1.28 mg) and E. carinatus (mean = 2.76 ± 0.35 mg) yielded much less **(table 1)**”, table 2 should be cited instead of table 1.

**Point 10:** Paragraph 3: “Biogeographic variation in venom yield was notable for *B. caeruleus*, with individuals from the Gangetic Plains producing substantially higher venom yields compared to those from the Western Ghats (Adjusted p = 0.0051; Figure 5A). In contrast, *N. naja*, *D. russelii*, and *E. c. carinatus* did not exhibit significant biogeographic differences in venom yield (Figures 3A, 4A, and 6A). Interestingly, sexual dimorphism did not seem to influence the venom yield either, which contrasts with some earlier studies that reported higher yields in females [12]. The distributions of venom yield for males and females of all four species overlapped considerably”. This feels more like a methods paragraph. Expand. Why do you think biogeographic variation was only observed for *B. caeruleus*?

**Point 11:** Paragraph 6: “*B. caeruleus* stands out for its exceptional potency”. Full species name should be used at the start of a sentence.

**General comments**

**Point 12:** The overall readability needs improving. Sentences should be improved for flow and clarity. I provide some examples below.

- **In Introduction (first paragraph):** “The so-called "big four" snake species, namely spectacled cobra (*Naja naja*), Russell’s viper (*Daboia russelii*), common krait (*Bungarus caeruleus*), and saw-scaled viper (*Echis carinatus*), are said to be responsible for most envenomations and snakebite-related morbidities and fatalities”.
  - Rephrase to: “The so-called "big four" snake species **include the** spectacled cobra (*Naja naja*), Russell’s viper (*Daboia russelii*), common krait (*Bungarus caeruleus*), and saw-scaled viper (*Echis carinatus*), **and** are responsible for most envenomations and snakebite morbidities and fatalities”
- **In Methodology (section 2.2):** “Experienced herpetologists employed safe handling methods, using acrylic restraining tubes to manually restrain **them**”.
  - Change “them” to “snakes”.
- **In discussion (second paragraph):** “while *B. caeruleus* (mean = 8.95 ±1.28 mg) and E. carinatus (mean = 2.76 ± 0.35 mg) yielded **much less** (table 1)”.
  - “Much less” is not appropriate for scientific writing.
- **In discussion (fourth paragraph): “**Adults produced the highest median yields, while subadults and juveniles yielded **much less** (Supplementary Table 2). For example, adult *N. naja* had a median yield of 125 mg, compared to 47.6 mg for subadults and **just** 6.5 mg for juveniles”.
  - “Much less” is not appropriate for scientific writing. Furthermore “and just 6.5” should be rephrased to “and 6.5”. “Just” is unnecessary.

**Point 13:** In Supplementary Table 2, second row, the venom yield for *N. naja* is 144. However, summing all the biogeography rows equals 143. Does this mean one sample is from an unknown locality? Conversely, summing the gender rows equal 106. Does this mean 38 samples were unknown gender? If yes, note this in the table caption. E.g. for some samples locality, gender, or age were not recorded.
