# Supplementary material for: Hiss and tell: What influences venom yields of India’s big four snakes?
Source: PLoS Negl Trop Dis. 2025 Nov 3;19(11):e0013676. doi: 10.1371/journal.pntd.0013676 (PMC12591399; doi:10.1371/journal.pntd.0013676)
Supplement: Attachment — Submitted filename: Response to Reviewers.docx [file pntd.0013676.s009.docx]

**Response to Reviewers**

**Manuscript ID:** PNTD-D-25-01566

**Title:** Hiss and tell: What influences venom yields of India's big four snakes?

**Reviewer #2:** Materials and methods

Seasonality and environmental covariates: In this study, years of collection (2021–2024) are available but not analyzed. Adding month/season to the model would visualize seasonal yield distributions by species. This could reveal phenology-linked dynamics of venom yield.

Thank you for this valuable suggestion. While we do have data for 2021-2024, our sampling strategy was determined by pragmatic features like ethical approvals, species availability, and logistical field constraints. Consequently, sampling was done opportunistically, as opposed to a seasonally organised design. Thus, we lack adequate and balanced sample sizes within months or seasons to carry out statistical analysis on the seasonal dynamics of venom yield. We recognise this as a limitation (now acknowledged in the discussion section) and emphasise that future studies with focused seasonal sampling will be necessary to gauge the effect of seasonality on venom yields.

**Reviewer #3:** - Although the objective is clearly stated, the methodology must be complemented with, at least, the HPLC/electrophoretic venoms profile, biochemical, enzymatic and in-vivo venoms analysis (proteomics also may be useful). The venom yielded by itself (and alone) is not worthy to be published as a full research article.

Thank you for your helpful recommendation. We acknowledge the importance of complementary analyses such as HPLC/electrophoretic venom profiling, biochemical, enzymatic, and in vivo studies, as well as proteomic characterization. However, these aspects have already been comprehensively addressed and published in previous works from the lab [(Senji Laxme et al. 2019; Sunagar et al. 2021; Senji Laxme et al. 2021; Senji Laxme et al. 2021; Senji Laxme et al. 2022; Jaglan et al. 2023; Senji Laxme et al. 2024; Rashmi et al. 2024; Sarangi et al. 2025; Javed et al. 2025; Rudresha et al. 2025)](https://paperpile.com/c/AcZUAz/o63Q+MDeK+k1DE+iCGa+yfJq+UOxE+OhZY+bVNV+wL6L+Vome+pChn). In the present study, we focus specifically on the determinants of venom yield in wild populations of Indian snakes—a previously underexplored area—as this ecological and quantitative information provides critical insights for antivenom manufacturers and clinicians.

**Reviewer #2:** Statistical reporting for the Kruskal-Wallis test on D. russelli geographic variation (p=0.011), the post-hoc test found no significant pairs. This should be briefly discussed in the results text, as it suggests that while overall variation exists, specific regional pairwise differences are not stark enough to show significance at multiple-testing correction.

Thank you for your suggestion. We have now added this explanation: “venom yield in *D. russelii* (n = 115; mean = 106.60 mg, median = 86.14 mg, range: 0.50–749.00 mg) varied significantly across India’s biogeographic zones (Kruskal–Wallis χ² = 13.05, df = 4, p = 0.011; Figure 3B), but post hoc Dunn’s tests with multiple comparison correction did not find significant pairwise differences (adjusted p>0.05).”

**Reviewer #3:**

1. Figure 1A. Please offer details about the approximately collecting areas. Where was collected each snake? India is such a huge and geographically diverse country and each of the showed biogeographic may include different ecogeographic compositions. Additionally, use colors to indicate which species was collected over each biogeographic area.

Thank you for this suggestion. The exact sample locations of each venom sample are now shown in Figure 1.

2. On table 1, “NA” means no specimens were collected? If so, please just add 0 (zero) in the table. The letters NA are not suitable for quantification purposes.

Thank you for pointing this out. We have revised Table 1 by replacing “NA” with “0” to indicate where no specimens were collected. However, we have retained NA for species that are ‘not available’ in the region. This has now been clarified in the legend.

3. Table 2 vs table 1 shows a difference on the number of D. russelii and B. caeruleus individuals collected and analyzed. Please correct.

Thank you for noticing this discrepancy. We have carefully rechecked the data and corrected the numbers.

4. On table 2 please decide to use one or two significative figures. After deciding, unify with the text.

Thank you for pointing this out. We have decided to use two significance digits in Table 2 and have unified this format consistently throughout the text.

5. On figure 4, “A” does not refer to “total yield” as stated in the figure foot note. It corresponds to the variation among the geographical region. B and C also do not correspond to what is shown in the figure. Finally, there is not figure D. Please correct.

Thank you for pointing this out. We apologise for the oversight. We have corrected the errors in Figure 4 to ensure that each panel is accurately described.

6. Figure 2B is not mentioned in the text. It must be.

Thank you for pointing out this oversight. We have now corrected this.

7. The text does not follow an order mentioning the figures. It goes from figure 2A to figure 4A. What happened (there is not mention of) with figures 2B, 2D, 3A, 3B and 3C? These kinds of gaps are not allowed according to the manuscript instructions of the PNTD (“Place figure captions in the manuscript text in read order”). <https://journals.plos.org/plosntds/s/figures>

Thank you for highlighting this issue. We have revised the manuscript to ensure that all figures are mentioned in proper sequential order.

8. The same as above is observed, in the same paragraph, jumping from figure 4A to figure 4B, the go back to figure 3A, move forward to figure 6A and finally go back to figure 2C and D. All in the same paragraph.

Thank you for pointing this out. We have revised the manuscript to correct the figure citation order and resolved the issue in the mentioned paragraph.

**Reviewer #2:** This manuscript represents important study in clinical toxinology. The data is robust, the analysis is sound, and the conclusions are well-supported and highly significant. The authors have provided a vital resource for improving snakebite management in India. The minor suggestions above are intended to further polish an already good manuscript.

We sincerely thank the reviewer for the positive and encouraging feedback. We have carefully addressed the minor suggestions to further improve the manuscript.

**Reviewer #3:** The manuscript does not present conclusions, so I will refer here to the discussion section.

1. In the discussion section, statements should be supported with the research data.

Thank you for your suggestion. We would like to clarify that the discussion section has been developed by directly integrating and interpreting research findings of this study. Each statement has been supported with data generated from the present study, along with relevant comparisons to previously published work. We have ensured that our interpretations remain closely aligned with the research data presented in the results.

2. In the discussion section the authors are refereeing to results not mentioned or obtained in their research.

Thank you for your feedback. We are unsure which specific section you were referring to. As mentioned above, the entire discussion is based on the findings obtained in this study or on comparisons with previously published data, which have been properly cited. Additionally, all related and supporting results like LD50 and comparison with previous data are included in the supplementary tables and figures. We have added additional information to these sections and hope that they address the reviewer’s concerns.

**Reviewer #3:** The authors present important and interesting information about the venom yielded by the big four from different regions emphasizing on the geographic, sex, age (life stage) and size. These results are particularly important since the venoms was obtained without applying external pressure. Unfortunately, the authors present only one result which should be complemented with other information about the venoms obtained. Please find below the major findings that doesn’t allows me to recommend these results for publication at the PNTD.

We sincerely thank the reviewer for feedback. We have carefully addressed the major suggestions to further improve the manuscript.

**Major findings.**

1. In the methodology section, the authors should clearly indicate the parameters used to classify the individuals as neonate, juvenile, subadult and adult.

Thank you for this comment. This has now been incorporated.

2. Results must be complemented with, at least, the HPLC/electrophoretic profile, biochemical, enzymatic and in-vivo analysis (proteomics also may be useful). The venom yielded by itself (and alone) is not worthy to be publisehd as a full research article.

Thank you for your suggestion. We acknowledge the importance of complementary analyses such as HPLC/electrophoretic venom profiling, biochemical, enzymatic, and in vivo studies, as well as proteomic characterization. However, these aspects have already been comprehensively addressed and published in previous works from the lab [(Senji Laxme et al. 2019; Sunagar et al. 2021; Senji Laxme et al. 2021; Senji Laxme et al. 2021; Senji Laxme et al. 2022; Jaglan et al. 2023; Senji Laxme et al. 2024; Rashmi et al. 2024; Sarangi et al. 2025; Javed et al. 2025; Rudresha et al. 2025)](https://paperpile.com/c/AcZUAz/o63Q+MDeK+k1DE+iCGa+yfJq+UOxE+OhZY+bVNV+wL6L+Vome+pChn). In the present study, we focus specifically on the determinants of venom yield in wild populations of Indian snakes—a previously underexplored area—as this ecological and quantitative information provides critical insights for antivenom manufacturers and clinicians.

3. The standard deviation (SD) is not a suitable parameter to indicate intraspecific variability.

Thank you for your comment. Because standard deviation (SD) is not a suitable parameter for intraspecific variability, we have also determined the Median Absolute Deviation (MAD), which is more robust to non-normal distributions. Additionally, we have assessed the Coefficient of Variation (CoV) to further characterise variability. These results have been included in the manuscript (Figure 6).

4. In the discussion section, statements should be supported with the research data.

Thank you for your suggestion. We would like to clarify that the discussion section has been developed by directly integrating and interpreting research findings of this study. Each statement has been supported with data generated from the present study, along with relevant comparisons to previously published work. We have ensured that our interpretations remain closely aligned with the research data presented in the results.

5. In the discussion section the authors are refereeing to results not mentioned or obtained in their research.

We are unsure which specific section you were referring to. As mentioned above, the entire discussion is based on the findings obtained in this study or on comparisons with previously published data, which have been properly cited. Additionally, all related and supporting results like LD50 and comparison with previous data are included in the supplementary tables and figures. We have added additional information to these sections and hope that they address the reviewer’s concerns.

**Reviewer #1:** The authors have presented a comparative study on venom yields and potency from the Indian “big four” snakes. Generally, the study provides new information with a concern of a very skewed sampling. However, it is worth noting that the authors have employed some statistical approaches to accommodate the skewedness. Additionally, the authors withheld a lot of information, especially in the introduction and methods. Finally, the study will add new information to the body of knowledge, and it is worth publishing if the authors address a few concerns raised below.

Introduction

Although the study is focused on the Indian “big four”, the introduction is scanty, just 273 words with only 3 references focusing on the main aims. The authors should consider introducing the nature of these variations as reported in other regions of the world, especially within Asia and within similar snake genera and species. This will inform the audience of the global aspect of potency and yield variations when it comes to snake venom.

We sincerely thank the reviewer for the positive and encouraging feedback.

Methods

The collection and measurement methods are so important in this study that it will be great if the authors add a stepwise protocol to the collection methods used and clarify how the collected venom was promptly measured before lyophilization. Additionally, the authors could clarify whether the same approach was used for the previously published study on snakes in captivity that was used for comparison

A detailed, stepwise protocol for venom collection methods has been included in the manuscript. We have clarified that the venom yield was measured before lyophilisation. We are unsure whether the same methodology was followed by the studies we are comparing our venom yields to. However, since we are comparing the dry weights of venom yields, pre‑lyophilisation volume measurements are not required for equivalence

Result

The results were well presented with clear figures. I will recommend that the authors consider adding “Supplementary Table 1: LD₅₀ values (mg/kg) of the ‘big four’ Indian snakes across biogeographic zones” as an adoption within the main manuscript since this is the main comparison of the potency which is part of the aim of the study. It should also contain information on the laboratory species in which the LD50 was determined.

Thank you for the kind words. We appreciate the reviewer’s observation. We have now moved Supplementary Table 1 to the main manuscript as Table 3. The strain of mice used for determining LD50s is clarified in the table legend.

Discussion

As mentioned earlier, it will be interesting for the audience to see how this Indian species differs or not compared with similar species found in other regions of the world especially in terms of potency and yield. Additionally, the authors focused more on the results than the implication of the results. It will be great if the authors will discuss further on how this new information will influence antivenom production, snakebite treatment, and the generally knowledge of these front fang snakes.

Thanks for your comments. We have now expanded the concluding paragraph to incorporate this suggestion.

**Reviewer #2:** Overall Assessment

This manuscript presents a pan-India analysis of venom yields from wild “big four” snakes (Naja naja, Daboia russelii, Bungarus caeruleus, Echis carinatus), spanning six biogeographic zones and 338 milking events. It documents inter/intraspecific and ontogenetic differences and contrasts wild yields with captive reports. The dataset is valuable and clinically relevant.

We sincerely thank the reviewer for their encouraging comments and recognition of the value and clinical relevance of our research.

**Reviewer #4:** The manuscript reports a comprehensive study of venom yields in India's "big four" medically important snakes (Naja naja, Daboia russelii, Bungarus caeruleus, and Echis carinatus). By analyzing dry venom yields from 338 wild-caught specimens, the authors found substantial variation between species. N. naja and D. russelii have the highest yields. Geographic differences in intra-species venom yield were significant only in B. caeruleus, between the Gangetic Plains and Western Ghats. The life stage of the snakes was found to be the major difference, with adults producing more venom than juveniles or subadults. Comparison with captive populations showed that captivity does not significantly alter venom yield.

This study addressed a significant gap in snakebite epidemiology and snake venom biology in India as it appears to be one of the most comprehensive studies on venom yields of a very large number of wild-caught “big four” medically important snakes. Given the burden of snakebite envenoming in India, the value and impact of this study cannot be understated.

We sincerely appreciate the reviewer's encouraging comments and their acknowledgment of the value and clinical relevance of our research.

I do not have any major concerns or comments, other than a few minor points as followed:

1. Since the major difference in venom yield was found to be life stages, I have to wonder if (intra-species) body size difference has been controlled for? As body weight/length was not among the reported data, I have to assume venom yield is not adjusted for body size. Can the authors comment (also in the manuscript) if body size is a confounding factor in when comparing juvenile/subadult vs adult snake venom yield?

Thank you for this insightful comment. We agree that body size is an important determinant of venom yield and could act as a confounding factor when comparing life stages. Unfortunately, morphometric data (e.g., body length and weight) were not consistently available for the wild-caught specimens due to forest department and government restrictions on handling these highly protected wildlife species in India. Instead, we relied on life-stage classifications based on size proxies and field expertise from herpetologists, which, while practical, are admittedly coarse approximations of body size. We acknowledge this as a limitation and have now clarified it in the revised manuscript. Despite this constraint, our relatively large sample size and consistent categorization across species provide robust support that the life stage is a primary driver of venom yield variation, even if body size effects cannot be fully disentangled. We have added this point in the discussion section for clarity.

2. While sample numbers for each category of factors can be found in Supplementary Table 2, it would be great if the information can also be provided in the graphs itself.

Thank you for your comment. We have added sample size information in all graphs as per your suggestions.

3. The last paragraph in page 5 and the first paragraph in page 6 (immediately below Table 1 legend) seems repetitive.

Thank you for your comment. The paragraph is revised in accordance with the heading to eliminate repetition within the manuscript.

4. Discussion: the second last paragraph discussed the correlations between venom yield with LD50. Obviously one of the major factors will be venom composition and the type of toxins present in the venom, Ie. common krait venom being predominantly neurotoxic will register a lower LD50 than the more hemotoxic venom of viper. Envenoming clinical presentation is also more complex than what LD50 can represent. Although the authors correctly point out there are multiple factors involved, can the authors expand this part of the discussion a little to present a more complete picture for the benefit of readers outside of the field.

Thank you for your suggestion. We have now incorporated this suggestion.

**Reviewer #2:** Overall Assessment

This manuscript presents a pan-India analysis of venom yields from wild “big four” snakes (Naja naja, Daboia russelii, Bungarus caeruleus, Echis carinatus), spanning six biogeographic zones and 338 milking events. It documents inter/intraspecific and ontogenetic differences and contrasts wild yields with captive reports. The dataset is valuable and clinically relevant.

We sincerely thank the reviewer for their encouraging comment.

Major Strengths

1. Large scale and ecological validity: The collection of 338 venom samples from wild populations across multiple biogeographic zones is a great effort. This provides data that is more ecologically relevant and representative of actual snakebite scenarios than previous studies reliant on captive snakes.

Thank you very much for your kind acknowledgement of our work.

2. Methodology: The use of non-parametric statistics is appropriate for the non-normally distributed data.

3. Clinically critical insights: The integration of venom yield data with known LD50 values to discuss envenomation strategies (e.g., high yield/moderate toxicity of N. naja vs. low yield/extreme potency of B. caeruleus) is a standout contribution. This directly informs risk assessment and it is important for snakebite treatment.

Recommendations

1. Introduction:

The introduction is concise and effective. The early statements in the first paragraph should be referenced

Thank you for your comment. We have now incorporated this suggestion.

2. Materials and methods

Seasonality and environmental covariates: In this study, years of collection (2021–2024) are available but not analyzed. Adding month/season to the model would visualize seasonal yield distributions by species. This could reveal phenology-linked dynamics of venom yield.

Thank you for this valuable suggestion. While we do have data for 2021-2024, our sampling strategy was determined by pragmatic features like ethical approvals, species availability, and logistical field constraints. Consequently, sampling was done opportunistically, as opposed to a seasonally organised design. Thus, we lack adequate and balanced sample sizes within months or seasons to carry out statistical analysis on the seasonal dynamics of venom yield. We recognise this as a limitation (now acknowledged in the discussion section) and emphasise that future studies with focused seasonal sampling will be necessary to gauge the effect of seasonality on venom yields.

3. Results

Statistical reporting for the Kruskal-Wallis test on D. russelli geographic variation (p=0.011), the post-hoc test found no significant pairs. This should be briefly discussed in the results text as it suggests that while overall variation exists, specific regional pairwise differences are not stark enough to show significance at multiple-testing correction.

Thank you for your suggestion. We have added this line in the manuscript:
“Similarly, although a statistically significant overall difference in venom yield was observed between males and females of the ‘big four’ Indian snakes (Mann-Whitney U = 6256, p = 0.0325; Figure 2C), sex-specific differences were not found within any species under investigation (adjusted p>0.05).”

4. Discussion

1. Our comprehensive and biogeographically inclusive assessment of venom yield in the pan-Indian populations of ‘big four’ snakes provides the first detailed insights into how ontogeny, sex, and geography affect venom production in wild populations of snakes.

Recast to read-

Our comprehensive and biogeographically inclusive assessment of venom yield in the pan-Indian populations of ‘big four’ snakes provides the first detailed insights into how ontogeny, sex, and geography affect venom yield in wild populations of snakes.

We thank the reviewer for the helpful suggestion. The sentence has been revised as recommended to improve clarity and consistency.

2. Adults produced the highest median yields, while subadults and juveniles yielded much less (Supplementary Table 2). For example, adult N. naja had a median yield of 125 mg, compared to 47.6 mg for subadults and just 6.5 mg for juveniles. – It is appropriate to compare the mean data in this instance rather than the median data.

We thank the reviewer for this suggestion. We chose to use median values instead of means because the venom yield data were not normally distributed and exhibited high variability. The median, therefore, provides a more robust measure of central tendency that minimizes the influence of outliers.

3. LD₅₀ values and venom yield data were analysed together to illustrate the distinct envenomation strategies of the big four snakes. B. caeruleus stands out for its

exceptional potency (LD₅₀ = 0.11 mg/kg) despite having the lowest average venom yield (9 mg). – The statement is misleading because from the results, E. c. carinatus had the lowest venom yield and not B. caeruleus

We thank the reviewer for pointing out this inconsistency. We agree that *E. c. carinatus* had the lowest venom yield, not *B. caeruleus*, as indicated in the results. We have corrected the statement in the Discussion section to accurately reflect the data. The revised sentence now reads:

“LD₅₀ values and venom yield data were analysed together to illustrate the distinct envenomation strategies of the big four snakes. *B. c. caeruleus* stands out for its exceptional potency (LD₅₀ = 0.11 mg/kg) despite having **one of the lowest average venom yield** (9 mg). In contrast, *N. naja* possessed moderate toxicity (LD₅₀ = 0.42 mg/kg) with the highest average venom yield (136 mg), which likely contributes to the higher rates of snakebite mortality caused by this species. Similarly, *D. russelii* produced very high venom yields (107 mg) and a comparatively low LD₅₀ (0.31 mg/kg). On the other hand, *E. c. carinatus* has the least toxic venom (LD₅₀ = 0.64 mg/kg) and the smallest venom yield (2.8 mg), yet remains a leading cause of envenomation.”

4. Overall, this research fills a critical gap in the understanding of venom yield variation in Indian snakes, highlighting the importance of size, geography, and life stage, while finding no evidence for the influence of sexual dimorphism.

Recast to read-

Overall, this research fills a critical gap in the understanding of venom yield variation in Indian snakes, highlighting the importance of size, geography, and life stage, while finding no evidence of sex-related differences

We thank the reviewer for this helpful suggestion. We agree that the revised phrasing improves clarity and readability.

“Overall, our research fills a critical gap in the understanding of venom yield variation in Indian snakes, highlighting the importance of size, geography, and life stage, while finding no evidence for the influence of **sex-related differences**.”

Conclusion

This manuscript represents a landmark study in clinical toxinology. The data is robust, the analysis is sound, and the conclusions are well-supported and highly significant. The authors have provided a vital resource for improving snakebite management in India. The minor suggestions above are intended to further polish an already good manuscript.

\# This study represents a major piece of work, with over 338 venom samples collected across India being used to investigate venom yields of India’s big four snakes. I commend the authors for such an extensive and important study. Venom yield is often overlooked in toxinology studies; thus, I believe this work is of high value to the scientific community, hopefully encouraging toxinologists to incorporate venom yields into their analyses.

Based off the novelty, extensive data collection, and applicability to future research, I recommend this research paper be accepted for publication pending major revisions.

We sincerely thank the reviewer for their extremely encouraging and positive feedback.

**Introduction**

**Point 1:** Although I commend the authors for having a concise introduction, it is too short. The introduction lacks impact and detail. I would like to see more literature on the activity of these snakes’ venoms across the biogeographic regions investigated in the study. What is the activity? How does it vary across regions? And most importantly, considering this variation, why is studying venom yield important for these snakes? Why might you expect yields to differ across biogeographic regions? Diet?

We thank the reviewer for this helpful suggestion. We have added this information in the introductory paragraph.

**Methods**

**Point 2:** “2.3 Statistical analysis”. A Supplementary material file of the statistical assumptions (e.g. normality tests) and outputs should be included. Additionally, another supplementary file containing all raw data should be provided, as well as the R scripts used to test effect sizes. Providing this will make the studies analyses completely transparent and reproducible.

We thank the reviewer for this helpful suggestion. We have now included a supplementary file 1 containing statistical code and the normality test result as per suggestion.

**Discussion**

**Point 3:** Paragraph 6: “These findings indicate that snakebite burden is shaped not just by venom potency alone, but by the interplay of potency, venom yield and other factors (Supplementary Tables 1 and 3).” This is a major finding and should not just be reserved for supplementary material. A figure and/or table showing the comparison between venom yield and LD50 should appear in the results section.

Thank you for this suggestion. We have now incorporated this suggestion.

**Introduction**

**Point 1:** The sentences: “India is home to an impressive variety of snakes, with over 426 species recorded to date. Among these, nearly 60 are clinically relevant to humans, requiring clinical intervention to prevent severe symptoms associated with their bites.”, needs a reference. Consider citing<https://reptile-database.reptarium.cz/>.

We thank the reviewer for this valuable suggestion. We have changed and updated the recent species count as per the [Mohapatra et al. 2024](https://paperpile.com/c/AcZUAz/jBD1) , i.e. ~350.

**Methods**

**Point 2:** “2.1 Ethical Statement”. This can be changed to “2.1 Snake venom samples”. Expand paragraph to include information on what species’ venom was collected and from where (biogeographic zone). Also include how many venom samples were collected (A total of 338). I know this information is in the authors’ supplementary data, but it fits better in the main paper. After reading this section, the reader should know the following main points: 338 individual snakes were milked encompassing four species, including male and female snakes; neonate, juvenile, subadult, and adult snakes, and snakes from different biogeographic regions.

Thank you for your suggestions. In the manuscript, we have now added section 2.1 'Ethical statement,' as you recommended. Additionally, we have incorporated your other suggestions into the revised manuscript.

**Point 3:** Animal samples (venom) were used in the study, thus reword the following sentence: “No animal or human samples were utilised in this study, and the LD50 values have been reported from our previous publications (Supplementary Table 1)”.

Thank you for your suggestions. We have revised the sentence in 2.1 Ethical statement as recommended.

**Results**

**Point 4:** In Figure 1B. the photo credits are missing. Please provide.

Thank you. Photo credits are now added.

**Point 5:** The paragraph starting with “Across all species and regions (N = 338 milking events),………..” should appear after Table 1.

Thank you for your comment. This section has been revised.

**Point 6:** The sentence: “Sex-specific differences were not found across any species under investigation (Figure 2C).”, needs rewording. Figure 2C shows a significant difference between males and females. I recommend the authors make it clear that although there is a significant difference overall between males and females, this difference was not observed within species.

Thank you for your comment. We have edited the manuscript as per your suggestion.

**Point 7:** The phrase “showing considerable overlap in their distributions.” is used throughout the methods to describe overlap of the data points between groups. Since differences in biogeographic regions are explored, the use of “distributions” may lead to confusion (distributions sounds like it is related to geography). Consider rewording.

Thank you for your suggestion. We have made changes as per your suggestion in the manuscript. “Distribution” is replaced by the “datapoints”.

**Discussion**

**Point 8:** The sentences: “Snakes were captured from the wild for sample collection and then safely released back at their original locations. This approach proved highly effective and non-harmful, as demonstrated by the recapture of a previously marked *E. carinatus* individual in the same area in subsequent years.” Would fit better in the methods section. Additionally, a reference is needed.

We feel this fits best in the results section. There is no reference to this as this is being reported here.

**Point 9:** In discussion (second paragraph) “while *B. caeruleus* (mean = 8.95 ±1.28 mg) and E. carinatus (mean = 2.76 ± 0.35 mg) yielded much less **(table 1)**”, table 2 should be cited instead of table 1.

Thank you for your comment. We resolved this problem.

**Point 10:** Paragraph 3: “Biogeographic variation in venom yield was notable for *B. caeruleus*, with individuals from the Gangetic Plains producing substantially higher venom yields compared to those from the Western Ghats (Adjusted p = 0.0051; Figure 5A). In contrast, *N. naja*, *D. russelii*, and *E. c. carinatus* did not exhibit significant biogeographic differences in venom yield (Figures 3A, 4A, and 6A). Interestingly, sexual dimorphism did not seem to influence the venom yield either, which contrasts with some earlier studies that reported higher yields in females [12]. The distributions of venom yield for males and females of all four species overlapped considerably”. This feels more like a methods paragraph. Expand. Why do you think biogeographic variation was only observed for *B. caeruleus*?

Thank you for your suggestion. We have revised as suggested. While plausible mechanisms can be suggested, attributing definitive causes would require targeted data and analyses beyond the scope of this study; accordingly, we provide only cautious hypotheses.

**Point 11:** Paragraph 6: “*B. caeruleus* stands out for its exceptional potency”. Full species name should be used at the start of a sentence.

Thank you for this suggestion. We have resolved this issue in the manuscript.

**General comments**

**Point 12:** The overall readability needs improving. Sentences should be improved for flow and clarity. I provide some examples below.

**In Introduction (first paragraph):** “The so-called "big four" snake species, namely spectacled cobra (*Naja naja*), Russell’s viper (*Daboia russelii*), common krait (*Bungarus caeruleus*), and saw-scaled viper (*Echis carinatus*), are said to be responsible for most envenomations and snakebite-related morbidities and fatalities”.

Rephrase to: “The so-called "big four" snake species **include the** spectacled cobra (*Naja naja*), Russell’s viper (*Daboia russelii*), common krait (*Bungarus caeruleus*), and saw-scaled viper (*Echis carinatus*), **and** are responsible for most envenomations and snakebite morbidities and fatalities”

We have rewritten many paragraphs in the paper. We have now incorporated this suggestion. Instead of ‘are responsible’ we would like to retain ‘are said to be responsible’ as a large number of bites are also contributed by clinically relevant yet neglected snake species in many regions.

**In Methodology (section 2.2):** “Experienced herpetologists employed safe handling methods, using acrylic restraining tubes to manually restrain **them**”.

Change “them” to “snakes”.

We appreciate the reviewer's suggestion. This has now been revised.

**In discussion (second paragraph):** “while *B. caeruleus* (mean = 8.95 ±1.28 mg) and E. carinatus (mean = 2.76 ± 0.35 mg) yielded **much less** (table 1)”.

“Much less” is not appropriate for scientific writing.

We thank the reviewer for this suggestion. We have revised the sentence to use more precise scientific language: “lower amounts of venom”

**In discussion (fourth paragraph): “**Adults produced the highest median yields, while subadults and juveniles yielded **much less** (Supplementary Table 2). For example, adult *N. naja* had a median yield of 125 mg, compared to 47.6 mg for subadults and **just** 6.5 mg for juveniles”.

“Much less” is not appropriate for scientific writing. Furthermore “and just 6.5” should be rephrased to “and 6.5”. “Just” is unnecessary.

We thank the reviewer for this suggestion. We have revised the sentence as suggested.

**Point 13:** In Supplementary Table 2, second row, the venom yield for *N. naja* is 144. However, summing all the biogeography rows equals 143. Does this mean one sample is from an unknown locality? Conversely, summing the gender rows equal 106. Does this mean 38 samples were unknown gender? If yes, note this in the table caption. E.g. for some samples locality, gender, or age were not recorded.

We thank the reviewer for noticing this. Indeed, some samples had missing metadata for locality, gender, and/or age. We have updated the caption of the supplementary table to clarify this.
